# Severe malaria enforces short-lived effector cell differentiation but does not prevent effective secondary responses by memory CD8 T cells

**DOI:** 10.1371/journal.ppat.1012993

**Published:** 2025-03-31

**Authors:** Jacob A. Hildebrand, Noah R. Daniels, Emma M. Dehm, Benjamin D. Fisher, Joseph K. Guter, Chris J. Janse, Erin D. Lucas, Jules A. Sangala, Trevor N. Tankersley, Geoffrey T. Hart, Sara E. Hamilton

**Affiliations:** 1 Center for Immunology, University of Minnesota, Minneapolis, Minnesota, United States of America; 2 Department of Laboratory Medicine and Pathology, University of Minnesota, Minneapolis, Minnesota, United States of America; 3 Leiden Malaria Research Group, Department of Parasitology, Center for Infectious Diseases, Leiden University Medical Center, Leiden, The Netherlands; 4 Division of Infectious Disease and Internal Medicine, Department of Medicine, University of Minnesota, Minneapolis, Minnesota, United States of America; University of Manchester, UNITED KINGDOM OF GREAT BRITAIN AND NORTHERN IRELAND

## Abstract

Parasitic infections are a major worldwide health burden, yet most studies of CD8 T cell differentiation focus on acute viral and bacterial infections. To understand effector and memory CD8 T cell responses during erythrocytic malaria infection in mice, we utilized transgenic OT-I T cells and compared CD8 T cell responses between infection with OVA-expressing strains of *Listeria monocytogenes* (Lm) and *Plasmodium berghei* ANKA (PbA). We find that CD8 T cells expand vigorously during both infections. However, in contrast to Lm infection, PbA infection induces T cells that are heavily biased toward an IL-7Ra-deficient and KLRG1+ short-lived effector cell (SLEC) phenotype at the expense of memory precursor effector cell (MPECs) formation. PbA-induced inflammation, including IFNγ, is partially responsible for this outcome. Following treatment with antimalarial drugs and T cell contraction, PbA-primed memory T cells are rarely found in the blood and peripheral tissues but do maintain a low presence in the spleen and bone marrow. Despite these poor numbers, PbA memory T cells robustly expand upon vaccination or viral infection, control pathogen burden, and form secondary memory pools. Thus, despite PbA enforced SLEC formation and limited memory, effective secondary responses can still proceed.

## Author summary

Following pathogen infection, CD8 T cells eliminate infected cells and form diverse pools of memory T cells that are critical to prevent reinfection. Both the timing and intensity of inflammatory signals present during T cell activation play a major role in the subsequent phenotype of responding CD8 T cells. Infection with *Plasmodium*, the causative agent of malaria, is a major worldwide health burden, but the response of CD8 T cells to this infection is not completely understood. Infection can result in a highly inflammatory and systemic infection of the blood. Using mouse models of infection, we asked how *Plasmodium* infection impacts responding CD8 T cells and compared this response to that of a well-studied bacterial infection, *Listeria monocytogenes*. We find that *Plasmodium* programs a unique phenotype among effector CD8 T cells that is at least partially caused by malaria-induced inflammation. Additionally, we find that after infection is resolved through antimalarial drug administration, CD8 T cells form substantially diminished memory pools. Despite this apparent deficiency, the few memory CD8 T cells that do persist are capable of expansion and protection following secondary infection. Our study highlights the unique impact of *Plasmodium* infection on CD8 T cells, and that seemingly poor memory does not necessarily impede host protection.

## Introduction

Following acute infection, effector CD8 T cells differentiate into two dominant subsets, short-lived effector cells (SLECs) and memory precursor effector cells (MPECs). Most studies focused on effector and subsequent memory CD8 T cell responses have utilized acute or chronic bacterial or viral infections, while the response to intracellular parasites remains understudied. Furthermore, the factors determining which effector cells survive into the memory phase are not fully understood and likely involve a complex array of signals, many of which are infection-specific. Here, we compared CD8 T cell differentiation and memory formation between two infections: *Listeria monocytogenes* (Lm) and *Plasmodium berghei* ANKA (PbA). Lm is an acute intracellular bacterium that has been used for decades as a model to study protective CD8 T cell responses [1]. PbA induces severe blood-stage malaria infection, and is used to study experimental cerebral malaria which is driven by CD8 T cell immunopathology [2].

Effector CD8 T cells are partitioned into MPEC and SLEC subsets based on the expression of killer-cell lectin-like receptor G1 (KLRG1) and the interleukin 7 receptor alpha chain (IL-7Rα). MPECs express high levels of IL-7Rα and low levels of KLRG1, while SLECs bear opposite expression of each marker. Previous work in the field established that the extent of inflammation present is the primary factor influencing whether CD8 T cells adopt an MPEC or SLEC fate [3]. For example, following acute viral infection, IL-12 signaling increases expression of the transcription factor T-bet. Genetic knockout of T-bet leads to a loss of SLECs, and overexpression of T-bet leads to more SLECs at the expense of MPECs [4]. Likewise, both type I and type II interferon signaling are necessary for SLEC differentiation and loss of either biases effector CD8 T cells towards an MPEC fate [5–7]. Together, these results demonstrate that effector T cell differentiation is closely tied to key inflammatory signals present during initial T cell expansion.

As infection resolves, MPECs give rise to most long-lived memory CD8 T cells, while the majority of SLECs die after the resolution of infection. However, our lab [8–10] and others [11] defined a long-lasting subset of KLRG1^+^ cells that persist through the contraction phase and join the memory pool. So-called long-lived effector cells (LLECs) exert greater pathogen control than other memory T cell subsets, despite their reduced ability to proliferate [8,9,11]. We have found that the proportion of LLECs present during the memory phase differs between infections, even when T cells of the same specificity are analyzed [9]. This suggests that environmental factors such as the duration of infection or level of inflammation may influence both effector CD8 T cells and the phenotype of the resulting memory CD8 T cell pool. We sought to investigate this further using the PbA mouse model, which induces a potent inflammatory response [12].

Malaria remains a significant global disease, with an estimated 250 million cases and 600,000 deaths annually [13]. *Plasmodium* infections occur in two stages, a hepatic stage in which the parasite reproduces in the liver, and an erythrocytic stage where the parasite causes systemic infection of red blood cells (RBCs). During the hepatic stage of malaria, memory CD8 T cells have been shown to confer sterilizing immunity in both mice and non-human primate models, and are thought to contribute to vaccine-induced immunity in humans [14].

During erythrocytic malaria, CD8 T cell contribution to the immune response against *Plasmodium* species is more complicated. Infected erythrocytes are filtered from circulation by phagocytes in the spleen, and CD8 T cells are primed through cross-presentation by CD8α+ dendritic cells, leading to expansion of effector T cells [15,16]. Because erythrocytes downregulate expression of major histocompatibility complex class I (MHC-I) during erythropoiesis, it was previously thought that CD8 T cells could only act in a “helper” role, by secreting key inflammatory molecules such as interferon gamma (IFNγ) [17]. However, during infection with *Plasmodium vivax* [18–20] or *Plasmodium yoelii* [21,22], it was recently shown that the parasite can infect immature erythroblasts that still express MHC-I, allowing CD8 T cells to eliminate infected erythrocytes in an antigen-specific manner. Whether this is a shared occurrence across all *Plasmodium* species remains to be tested. Additionally, CD8 T cells can cause immunopathology in the brain and lungs of mice upon recognition of cross-presented antigen on blood endothelial cells [23–26], and recent evidence has implicated a role for CD8 T cells during human cerebral malaria [27].

Given the diverse roles CD8 T cells play during *Plasmodium* infections, and the unique route of antigen presentation and high level of systemic inflammation present during priming, we sought to characterize the effector and memory CD8 T cell response to PbA infection. We find that in contrast to the T cell response following Lm infection, PbA infection programs an effector T cell pool that is almost exclusively comprised of SLECs. After PbA infection resolves through anti-malarial treatment, contraction leads to <1% of antigen-specific T cells persisting in the blood or peripheral non-lymphoid tissues while a small population is preserved in the spleen and other lymphoid tissues. Despite the strong SLEC phenotype, most remaining memory T cells adopt a central memory phenotype rather than becoming LLECs. We find that the SLEC bias is at least partially due to inflammation, as global deletion of IFNγ allows for changes in effector T cell phenotypes. Additionally, bystander coinfection with malaria was sufficient to reduce the size of the memory T cell pool. Despite the reduced presence of memory CD8 T cells in the periphery following PbA infection, the remaining cells can expand more than 10^3^-fold in response to vaccination or viral infection and effectively reduce viral load.

In conclusion, our data support a model in which PbA infection induces memory CD8 T cells that are highly resilient despite disruptions in conventional T cell effector differentiation, remaining capable of long-term persistence and protection. Thus, infections which heavily bias toward SLEC formation do not preclude the host from forming a diverse and effective memory pool.

## Results

### OT-I T cells responding to PbA infection are phenotypically similar to responding endogenous T cells

Although *Plasmodium* epitopes recognized by mouse CD8 T cells have been identified, the introduction of TCR transgenic T cells recognizing epitopes like ovalbumin (OVA) enables probing of the same T cell specificity across different types of infections. Before using transgenic OT-I T cells to study CD8 T cell responses to PbA, we verified that these cells were phenotypically and functionally similar to endogenous T cells responding to a *Plasmodium* antigen. PbA infection generates CD8 T cells that recognize the glideosome-associated protein 50 (GAP50) TCR epitope [23], which we identified by staining with GAP50_41-48_/D^b^ MHC tetramers.

Mice receiving OT-I T cells were infected with *Plasmodium berghei* ANKA parasites with constitutive expression of mCherry-OVA (PbA-OVA) [28], and cells from various tissues were analyzed by flow cytometry on day 7 post-infection (p.i.). Both transgenic OT-I T cells and endogenous GAP50^+^ cells were detectable in the spleen and peripheral tissues at day 7 p.i. (S1A Fig), with OT-I T cells present at ~20-fold higher numbers than GAP50+ cells. Despite a numerical difference, the localization and phenotype of OT-I T cells and endogenous T cells were similar. Using intravascular labeling [29], we found that most OT-I and GAP50^+^ T cells in the spleen were confined to the white pulp, while most cells in the periphery were found in the vasculature (S1B Fig). OT-I and GAP50^+^ T cells in the blood, lungs, and brain were highly biased toward a KLRG1^+^ SLEC phenotype, rather than a KLRG1^-^ MPEC phenotype (S1C Fig). Additionally, OT-I and GAP50^+^ T cells in the blood, lungs and brain expressed high levels of IL-2, IFNγ, and granzyme B directly *ex vivo* (S1D Fig), suggesting that these cells were recently exposed to antigen in these tissues. Together, these data indicate that OT-I T cells expand robustly during infection and are phenotypically, functionally, and located similarly to endogenous *Plasmodium*-specific CD8 T cells.

### Expansion of OT-I T cells following Lm or PbA infection leads to two distinct differentiation patterns

**Fig 1:**
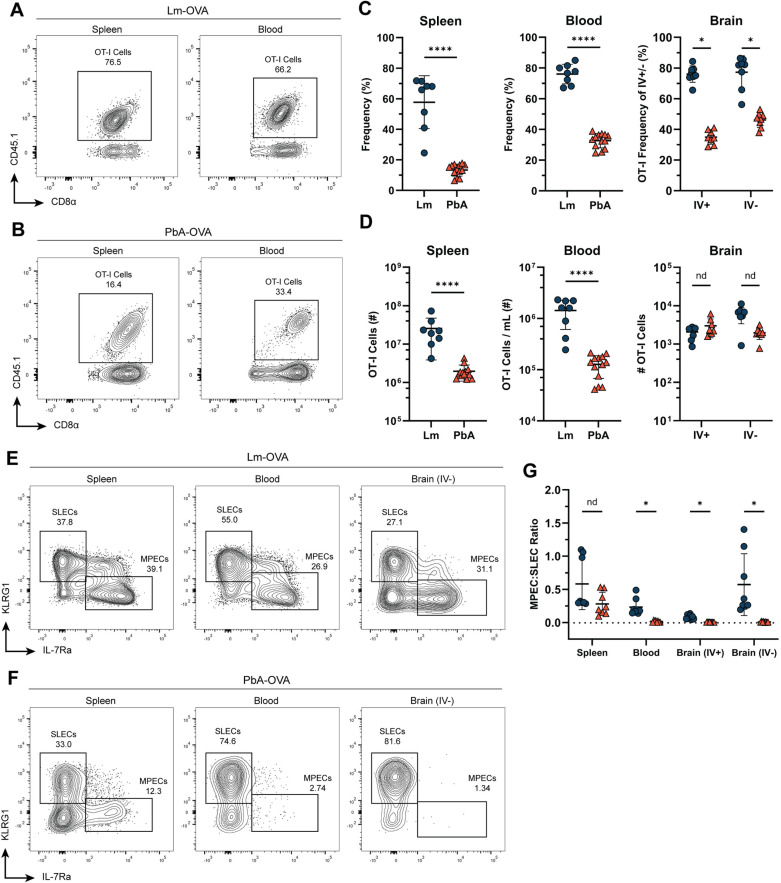
Expansion of OT-I T cells following Lm or PbA infection leads to two distinct differentiation patterns. (A and B) Representative flow cytometry plots of CD45.1^+^ OT-I T cells in the spleen and blood on day 7 p.i. with either *Listeria monocytogenes* expressing OVA (Lm-OVA) (A) or *Plasmodium berghei* ANKA expressing OVA (PbA-OVA) (B). (C) Frequency of CD45.1^+^ OT-I T cells in the indicated tissues on day 7 p.i. with either Lm-OVA (blue) or PbA-OVA (orange). Values represent the frequency of CD45.1^+^ cells among the responding (CD44^+^) effector T cell pool. (D) Total number of CD45.1^+^ cells in the indicated tissues on day 7 p.i. with either Lm-OVA or PbA-OVA. (E and F) Representative KLRG1 x IL-7Rα flow cytometry plots showing the effector phenotype of OT-I T cells in the indicated tissues on day 7 p.i. with Lm-OVA (E) or PbA-OVA (F). (G) Ratio of IL-7Rα^hi^ KLRG1^-^ memory precursor effector cell (MPEC) phenotype to IL-7Rα^lo^ KLRG1^+^ short-lived effector cell (SLEC) phenotype after gating on CD45.1^+^ OT-I T cells in the indicated tissues on day 7 p.i. with either Lm-OVA or PbA-OVA. Data in A-D is pooled from three independent experiments with ****n**** = 3-5 mice per group and analyzed with a Mann-Whitney test. Data in E-G is pooled from two independent experiments with ****n**** = 3-5 mice per group. *p < 0.05, **p < 0.01, ***p < 0.001, ****p < 0.0001. Error bars are SD. See also S1 and S2 Figs.

Next, we compared the number and phenotype of OT-I T cells on day 7 p.i. between PbA-OVA infected mice and those infected with *Listeria monocytogenes* expressing OVA (Lm-OVA). Mice infected with Lm-OVA had a significantly greater proportion and number of OT-I T cells when compared to PbA-infected mice at this time point (Figs 1A–1D & S2C–S2F). Intriguingly, despite a large difference in cell number in other tissues, both infections recruited a similar number of cells to the vasculature of the brain and lungs (Figs 1D & S2D). Relative to the spleen, a significantly higher ratio of cells was present in the IV+ fraction of both tissues following PbA-OVA infection (S2G Fig) when compared to Lm-OVA. Both the lungs and brain experience immunopathology following infection with PbA[23,26], and these results further support tissue-specific tropisms during this infection. The localization of OT-I T cells responding to either infection was similar in most peripheral tissues, although there were significantly more OT-I T cells in the splenic red pulp of PbA-infected mice (S2H Fig).

The most striking difference between Lm-OVA- and PbA-OVA-infected mice was the phenotype of responding effector cells. OT-I T cells responding to Lm-OVA held a slight bias towards KLRG1^+^ SLECs but retained a well-defined population of IL-7Rα^hi^ MPECs in all tissues (Fig 1E & 1G). These results align with other Lm studies of either endogenous or transgenic CD8 T cells [7,30]. Conversely, OT-I T cells responding to PbA-OVA were heavily biased toward a SLEC phenotype in all sampled tissues except the spleen, with fewer cells expressing IL-7Rα (Fig 1F & 1G). This contrasts with other acute viral [4,7,31] and parasitic infections [32,33], including infection with *Plasmodium yoelii* [20], which all produce effector populations with substantial frequencies of both SLECs and MPECs in various tissues. Our findings suggest that PbA infection uniquely inhibits MPEC formation, particularly in the blood and peripheral tissues.

### OT-I T cells form diminished memory following PbA infection

Although SLECs are more vulnerable to apoptosis during the contraction phase, we previously showed that LLECs derive almost exclusively from the SLEC effector pool and persist long term [9]. Furthermore, previous work using KLRG1-reporter mice has shown that KLRG1^+^ effector cells can lose expression of this molecule during the effector phase and join the central and effector memory pools [34]. Therefore, we sought to assess the number and phenotype of memory CD8 T cells following PbA infection. We speculated that the high frequency of SLECs during the effector phase would generate a memory T cell pool comprised primarily of LLECs.

**Fig 2:**
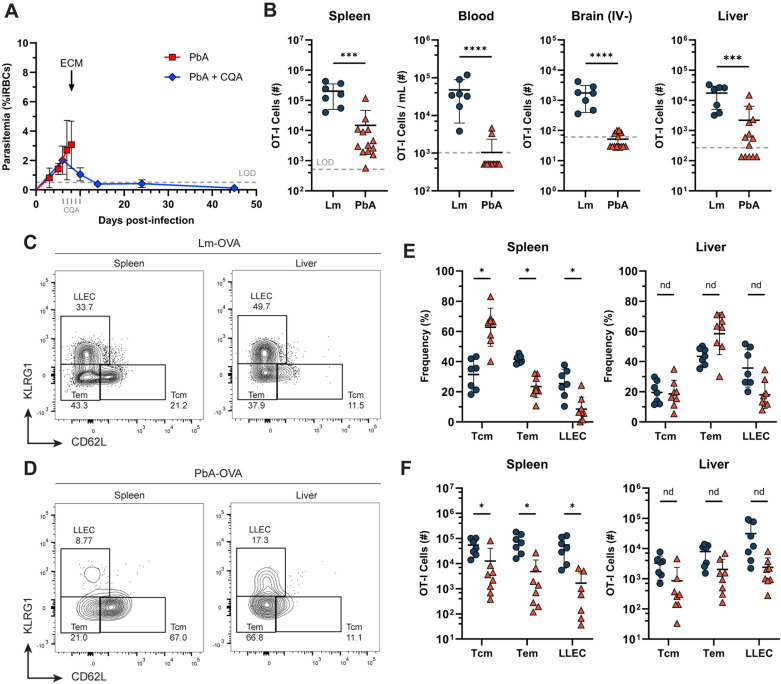
OT-I T cells form diminished memory following PbA infection. (A) Mice were infected with PbA-OVA, with one group of mice left untreated (red) and another group (blue) being treated with 30 mg/kg chloroquine and 30 mg/kg artesunate (CQA) to cure blood-stage malaria. Parasitemia was tracked in the blood on the indicated days post-infection until the development of cerebral disease (~D8 p.i.) or experimental endpoint (D45 p.i.). (B) Total number of OT-I T cells in the indicated tissues on day 45 p.i. with either Lm-OVA (blue) or PbA-OVA (orange). Gray line represents limit of detection (LOD), measured in S3D–E Fig. (C and D) Representative KLRG1 x CD62L flow cytometry plots showing the memory phenotype of OT-I T cells in the spleen and liver on day 45 p.i. after infection with Lm-OVA (C) or PbA-OVA (D). (E) Frequency of OT-I T cells bearing a KLRG1^-^ CD62L^+^ central memory (Tcm), KLRG1^-^ CD62L^-^ effector memory (Tem), or KLRG1^+^ CD62L^-^ long-lived effector cell (LLEC) phenotype in the spleen and liver on day 45 p.i. with either Lm-OVA (blue) or PbA-OVA (orange). (F) Total number of OT-I T cells with a Tcm, Tem, or LLEC phenotype in the spleen and liver on day 45 p.i. with Lm-OVA (blue) or PbA-OVA (orange). Data in A is pooled from two experiments with *n=* 4-7 mice per group. Data in B-D is pooled from three independent experiments with ****n**** = 3-5 mice per group and analyzed with a Mann-Whitney U test. Values below the LOD were set to LOD/2 and excluded from phenotypic analysis. Data in E-F is pooled from two independent experiments with ****n**** = 3-4 mice per group and analyzed with multiple Mann-Whitney tests. *p < 0.05, **p < 0.01, ***p < 0.001, ****p < 0.0001. Error bars are SD. See also S3 and S4 Figs.

Mice receiving OT-I T cells and PbA-OVA were treated with the antimalarial drugs chloroquine and artesunate daily from day 6 through day 10 p.i. to prevent the development of cerebral pathology [35]. Chloroquine inhibits malaria parasites by preventing the conversion of heme to hemozoin [36], while artesunate prevents nucleic acid synthesis during the erythrocytic stages of all *Plasmodium* species [37]. Parasitemia was monitored regularly via flow cytometry [38] (Fig 2A) to ensure clearance of the parasite. On day 45 p.i. the presence and phenotype of OT-I T cells was assessed via flow cytometry.

After Lm-OVA infection, OT-I T cells represented 10-20% of the CD8 T cells in the blood and spleen, with similar frequencies observed in most peripheral tissues (Figs 2B & S3A–S3C). OT-I T cells primed by Lm-OVA in the spleen and liver were evenly distributed between KLRG1^-^ CD62L^+^ central memory T cells (Tcm), KLRG1^-^ CD62L^-^ effector memory T cells (Tem), and KLRG1^+^ CD62L^-^ LLECs (Fig 2C–2F). In stark contrast, after PbA-OVA infection the proportion and number of OT-I T cells present was much lower (Fig 2B). While OT-I T cells were detectable in lymphoid tissues (Figs 2B & S3A–S3C), counts were below the limit of detection (S3D–S3E Fig) for the blood and peripheral tissues in most mice. OT-I T cells in the spleen were significantly biased towards Tcm when compared with Lm-primed mice, at the expense of both Tem and LLECs (Fig 2D–2F). In the liver, OT-I T cells were biased towards Tem, with a reduced frequency of Tcm and LLECs. Other tissues, including the blood, lung, and brain, all contained too few cells to determine a phenotype (S4A & S4B Fig).

We also examined additional tissues, including the inguinal lymph node, bone marrow, and salivary gland (Fig S3A–C). In the salivary gland, we again found few memory OT-I T cells in PbA-infected mice, while these cells were detectable in mice infected by Lm. Like the spleen, OT-I T cells were found in both the inguinal lymph node and bone marrow following PbA infection, although this population was still significantly lower than mice infected with Lm-OVA (S3A Fig). The phenotype of PbA-primed OT-I T cells in the lymph node was similar to the spleen, with slightly more Tcm cells at the expense of LLECs (S4A–D Fig). OT-I T cells in the bone marrow did not bear any phenotypic differences between Lm and PbA infection. Collectively, this data demonstrates that few OT-I memory cells form after PbA-OVA infection, in stark contrast to infection with Lm-OVA. The OT-I T cells that do persist tend to be in lymphoid tissues, with a bias towards Tcm rather than an LLEC phenotype.

### CD8 T cell memory is not inhibited by antimalarial treatment

**Fig 3:**
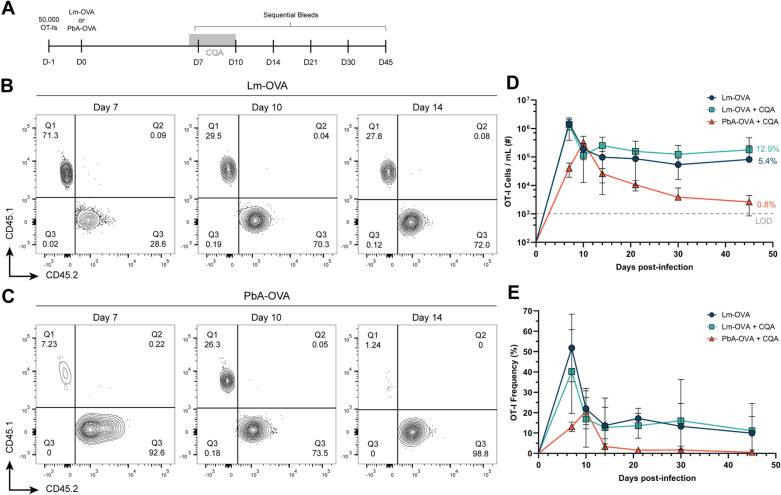
CD8 T cell memory is not inhibited by antimalarial treatment. (A) Experimental schematic: Mice were transferred with OT-I T cells before infection with either Lm-OVA or PbA-OVA. Three groups were prepared: Lm-OVA infected, Lm-OVA infected treated with 30 mg/kg chloroquine and artesunate (CQA) on days 6-10 p.i., PbA-OVA infected treated CQA on days 6-10 p.i. Mice were bled on days 3, 7, 10, 14, 21, 30, 45 p.i. (B and C) Representative flow cytometry plots showing CD45.1^+^ OT-I T cells on days 7, 10, and 14 p.i. with either Lm-OVA (B) or PbA-OVA (C). (D) The total number of OT-I T cells per mL of blood in Lm-OVA (blue), Lm-OVA + CQA (green), PbA-OVA + CQA (orange) groups was tracked over time. The numbers on the right indicate the percentage of cells remaining following T cell contraction after Lm-OVA infection (Day 45/ Day 7) or PbA-OVA infection (Day 45/ Day 10). (E) Frequency of OT-I T cells in the blood (gated on CD8^+^ CD44^+^ cells) over time following infection in the indicated groups. Data in B-E is pooled from four independent experiments with ****n**** = 3-5 mice per group. Error bars are SD. See also S5 Fig.

Chloroquine and artesunate (CQA) are highly effective antimalarial drugs, but also have a variety of immunomodulatory effects that could affect T cell persistence. Chloroquine interferes with lysosomes in monocytes and dendritic cells, which impairs antigen presentation and dampens CD4 T cell function [39–42]. Drugs in the artemisinin family such as artesunate can also inhibit immune cell activation, including T cells [43]. We considered whether the antimalarial regimen used to clear blood-stage infection was interfering with CD8 T cell maintenance into the memory phase. To test this, mice received CD45.1^+^ OT-I T cells, followed by infection with either PbA-OVA or Lm-OVA. A third group of mice were infected with Lm-OVA and treated with antimalarials alongside the PbA-infected group. The presence of OT-I T cells in the blood was then tracked over time (Fig 3A).

As expected, Lm-OVA infection led to a robust expansion of OT-I T cells and established stable cell numbers by day 14 that continued into the memory phase (Fig 3B & 3D–3E). Treatment with CQA did not alter the expansion or longevity of OT-I T cells in Lm-OVA infected mice, which was similar to untreated controls. Based on the blood collection time points in this experiment, the OT-I response to PbA-OVA peaked on day 10 p.i., followed by a striking contraction phase between days 10 and 14 p.i., and a steady decline in OT-I numbers over the next four weeks (Fig 3C–3E). It has been documented in multiple infection and vaccination regimens that CD8 T cells undergoing contraction reduce cell numbers by ~90%, while the remaining cells become stable CD8 T cell memory [44–48]. After contraction, Lm-infected mice maintain a steady frequency of 5-13% of their numerical peak into the memory phase (Day 45 compared to Day 7). On the contrary, OT-I T cells in PbA-infected mice only maintain 0.8% of their numerical peak (Day 45 compared to Day 10) (Fig 3D).

One possible explanation for the sharp contraction of OT-I T cells following PbA infection could be a form of T cell exhaustion. This is an active area of research during PbA infection, with some studies suggesting that T cell expression of inhibitory molecules among splenocytes during the effector phase is associated with higher function rather than exhaustion [49,50]. However, these studies did not track OT-I expression of inhibitory molecules longitudinally. To this end, we stained OT-I T cells for PD-1, CTLA-4, Lag3, Tim3, and Tox1 during Lm-OVA and PbA-OVA infection and tracked their expression in the blood. We find that as Lm-OVA primed and PbA-OVA-primed OT-I T cells undergo T cell contraction, their expression of canonical exhaustion markers remains low or absent (S5A–E Fig).

Another reason for the decline in OT-I T cells is that PbA infection might induce lymphopenia, which has been described in other highly inflammatory settings of systemic infection [51,52]. However, after a brief increase during the effector stage of each infection, the number of total CD4 and CD8 T cells in the blood remained constant in all three groups (S5F & S5G Fig). Collectively, these results indicate that the diminished T cell memory observed following PbA infection is not caused by antimalarial treatments or systemic lymphopenia, and that T cells do not express traditional markers of exhaustion.

### PbA infection inhibits CD8 T cell expression of IL-7Ra and limits T cell memory during bacterial coinfection

Both SLECs and MPECs are normally generated during the effector phase of infection, but outside of the spleen, we found few OT-I T cells with an MPEC phenotype during the effector phase of PbA infection (Fig 1F & 1G). IL-7 signaling is an essential component of T cell homeostasis [53], and the upregulation of IL-7R is typically observed as T cells transition from effectors to memory cells.

**Fig 4:**
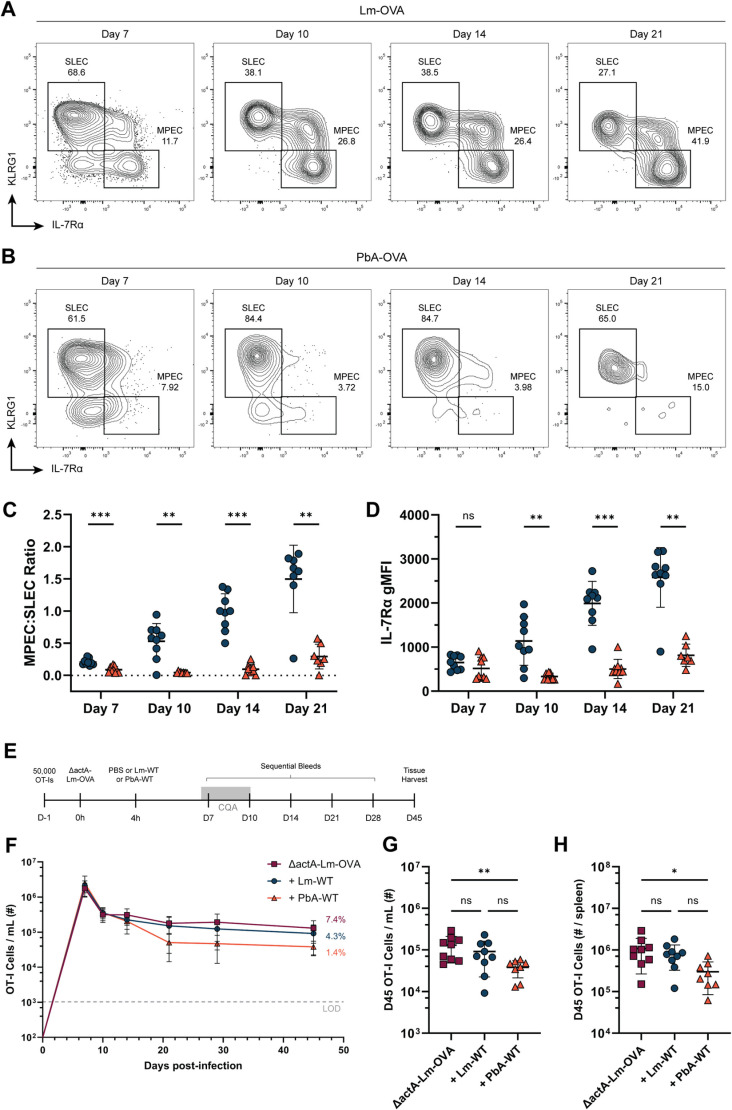
Severe PbA infection inhibits CD8 T cell expression of IL-7Ra. (A and B) Representative KLRG1 x IL-7Rα flow cytometry plots showing the effector phenotype of OT-I T cells in the blood on days 7, 10, 14, and 21 p.i. with Lm-OVA (A) or PbA-OVA (B). (C) The ratio of IL-7Rα^hi^ KLRG1^-^ MPECs to IL-7Rα^lo^ KLRG1^+^ SLECs after gating on CD45.1^+^ OT-I T cells on days 7, 10, 14, and 21 p.i. after infection with either Lm-OVA (blue) or PbA-OVA (orange). (D) The mean-fluorescence intensity of IL-7Rα on OT-I T cells on days 7, 10, 14, and 21 p.i. with Lm-OVA or PbA-OVA. (E) Experimental schematic for coinfection experiment: Mice receiving OT-I T cells were infected with ΔActA-Lm-OVA the following day. 4 hours after infection with ΔActA-Lm-OVA, mice were coinfected with either Lm-WT or PbA-WT, and sequential bleeds were performed out to memory. (F) Plot representing the total # of OT-I T cells/ mL of blood over time in the indicated groups. The numbers on the right indicate the percentage of memory T cells remaining after T cell contraction (Day 45/ Day 7). (G) The total number of OT-I T cells in the blood on day 45 p.i. in the indicated groups. (H) The total number of OT-I T cells in the spleen on day 45 p.i. in the indicated groups. Data in A-D is pooled from two independent experiments with ****n**** = 3-5 mice per group, and groups in C-D were analyzed with multiple Mann-Whitney tests. Data in F-G is pooled from two independent experiments with ****n**** = 3-5 mice per group. Data in G-H was analyzed with a one-way analysis of variance (ANOVA). *p < 0.05, **p < 0.01, ***p < 0.001 , ****p < 0.0001. Error bars are SD.

We tracked OT-I T cells in the blood of mice infected with either Lm-OVA or PbA-OVA through the effector, contraction, and memory phases. As expected, Lm-OVA infection generated more SLECs during the early stages of infection, but an increase in the proportion of MPECs as the cells contracted (Fig 4A & 4C). By comparison, OT-I T cells that were primed with PbA-OVA were heavily skewed towards a KLRG1^+^ SLEC phenotype on days 7, 10, and 14 p.i. with very few MPECs (Fig 4B & 4C). We measured the mean fluorescence intensity of IL-7Rα on OT-I T cells and found that it increased over time after Lm-OVA infection, with only a minimal change after PbA-OVA (Fig 4D).

The level of inflammation present during priming is a key determinant of CD8 T cell differentiation [4]. We sought to test whether PbA-induced inflammation alone was sufficient to alter OT-I effector differentiation and prevent formation of a robust memory T cell pool. To do this, we transferred mice with OT-I T cells and infected them with ΔActA-Lm-OVA, a less virulent strain of *Listeria monocytogenes* [54]. Four hours later, some mice were also coinfected with either Lm-WT or PbA-WT (neither expressing OVA) (Fig 4E). This experimental approach eliminated any differences in OT-I priming and kinetics that might exist between Lm-OVA and PbA-OVA, and directly tested whether PbA-induced inflammation affects T cell persistence.

During the first 10 days of infection, we observed identical expansion and contraction between all three groups (Fig 4F). Mice that were coinfected with ΔActA-Lm-OVA and Lm-WT maintained a similar level of OT-I T cells in the blood out to memory, with no difference in the number of OT-I T cells found per spleen (Fig 4G & 4H). Intriguingly, coinfection with PbA-WT led to a decline in OT-I T cell numbers by day 21 p.i., with significantly fewer cells found in the blood and spleen at memory (Fig 4G & 4H). While this experiment did not fully recapitulate the reduction in CD8 T cell memory during PbA-OVA infection, it shows that a bystander infection with PbA reduces T cell memory formation.

### Loss of interferon gamma improves MPEC formation but does not rescue persistence of PbA-primed OT-I T cells

Malaria infection induces a vigorous inflammatory response, including high amounts of systemic IFNγ [55–58]. In clinical settings of malaria, multiple studies have implicated atypical B cells [59,60] as responsible for the poor generation of humoral immunity against *Plasmodium* species. While a direct link to malaria-induced inflammation has not been made in humans, Ryg-Cornejo et al. found that PbA infection impairs germinal centers, and that blockade of IFNγ improved overall humoral immunity [61].

Based on our finding that PbA infection encourages a significant bias towards SLECs when compared to Lm infection, we asked whether removing just one source of inflammation during infection would change the phenotype or persistence of OT-I T cells. To test this, we infected either wild-type (WT) mice or IFNγ^-/-^ mice with PbA and tracked the number and phenotype of OT-I T cells in the blood over time. We first measured whether systemic loss of IFNγ affected parasitemia levels, which could alter the quantity and duration of antigen stimulation. Although parasitemia on day 6 post-infection was slightly elevated in IFNγ^-/-^ mice (1.2%) compared to WT mice (0.7%), this trend was not significant (S6A Fig). As a control group to track OT-I T cells longitudinally, we used ΔActA-Lm-OVA, as the loss of host IFNγ impairs the ability of mice to clear wild-type Lm-OVA infection [62]. We hypothesized that removal of IFNγ from the host would increase MPEC formation in both infections and improve the persistence of OT-I T cells after PbA infection.

**Fig 5:**
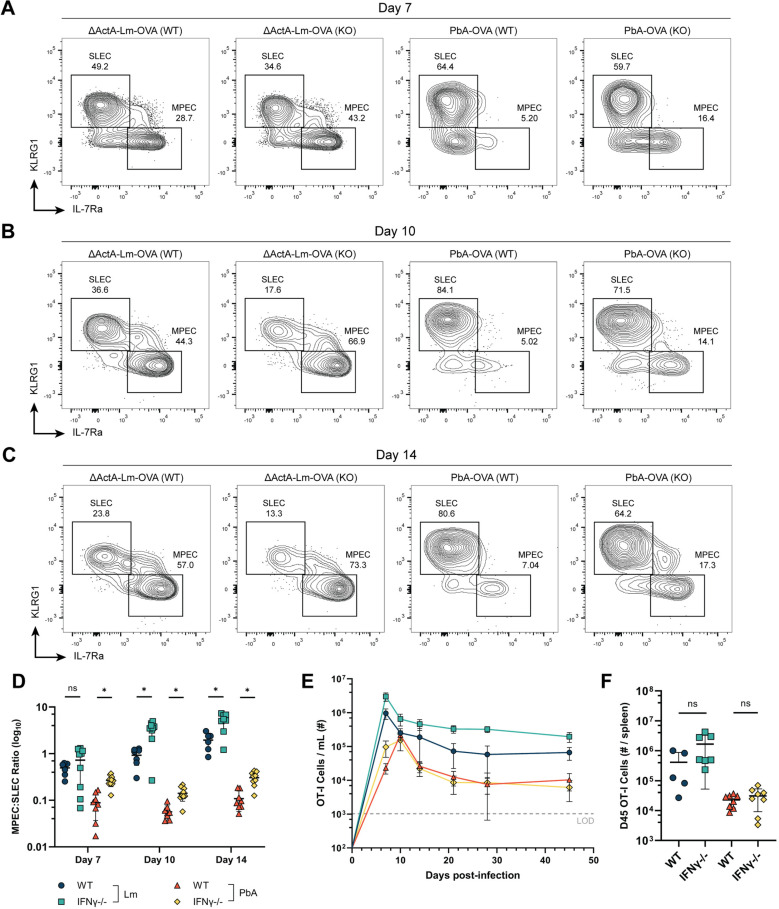
Loss of interferon gamma improves MPEC formation but does not rescue persistence of PbA-primed OT-I T cells. Wild-type (WT) and interferon gamma knockout (KO) mice were infected with either ΔActA-Lm-OVA or PbA-OVA. (A-C) Representative KLRG1 x IL-7Rα flow cytometry plots showing the effector phenotype of OT-I T cells in the blood on day 7 (A), day 10 (B), and day 14 (C) post-infection. (D) The ratio of MPECs to SLECs on days 7, 10, and 14 p.i. with either ΔActA-Lm-OVA or PbA-OVA in WT (blue and orange) or IFNγ KO (green and yellow) mice. (E) Plot representing the total # of OT-I T cells/ mL of blood over time after infection with either ΔActA-Lm-OVA or PbA-OVA in WT or IFNγ KO mice. (F) The total number of OT-I T cells in the spleen on day 45 p.i. with either ΔActA-Lm-OVA or PbA-OVA in WT or IFNγ KO mice. Data in A-F is pooled from three independent experiments with ****n**** = 2-5 mice per group. WT and IFNγ-/- mice were compared using multiple Mann-Whitney tests. *p < 0.05, **p < 0.01, ***p < 0.001, ****p < 0.0001. Error bars are SD. See also S6 and S7 Figs.

Consistent with our expectations, OT-I T cells primed by ΔActA-Lm-OVA infection in the absence of IFNγ were significantly biased towards MPECs when compared to OT-I T cells primed in WT mice (Fig 5A–5D). Following initial expansion, IFNγ deficiency led to significantly more OT-I T cells persisting to memory in Lm-OVA infected mice (Fig 5E), which aligns with previous reports for CD4 and CD8 T cells [62,63]. Strikingly, the phenotype of OT-I T cells responding to PbA-OVA was also affected by loss of IFNγ, with a clear increase in the frequency of MPECs in these mice (Fig 5D). However, this phenotypic change did not translate to improved persistence in PbA-infected mice, with a similar number of OT-I T cells present in the blood and spleen at memory (Fig 5E & 5F). Loss of IFNγ did alter the phenotype of memory OT-I T cells in the spleen in PbA-infected mice, with a reduced frequency of LLECs in IFNγ^-/-^ mice in favor of Tem (S6B–S6D Fig), suggesting a role for IFNγ during LLEC maintenance. While this data demonstrates an improvement in MPEC formation when IFNγ is removed, the total number of memory T cells did not increase, suggesting that additional signals contribute to the sharp contraction observed in PbA-infected mice.

To eliminate the possibility of wild-type OT-I T cells using autocrine IFNγ, we used CRISPR-Cas9 to delete IFNγ (cIFNγ) from OT-I T cells, with deletion of CD90 (cCD90) as a control. These OT-I T cells were then transferred into WT or IFNγ^-/-^ hosts, followed by Lm-OVA or PbA-OVA infection (S7A Fig). Following *in vitro* peptide stimulation, we found that our CRISPR-based approach significantly reduced IFNγ production by cIFNγ OT-I T cells compared to cCD90 controls (S7D & S7E Fig). Additionally, we find that regardless of whether systemic IFNγ is present in the host, the effector phenotype of OT-I T cells is not affected by loss of autocrine IFNγ signaling (S7F–S7H Fig).

### PbA-primed OT-I memory cells expand following peptide vaccination and provide protection against viral infection

**Fig 6:**
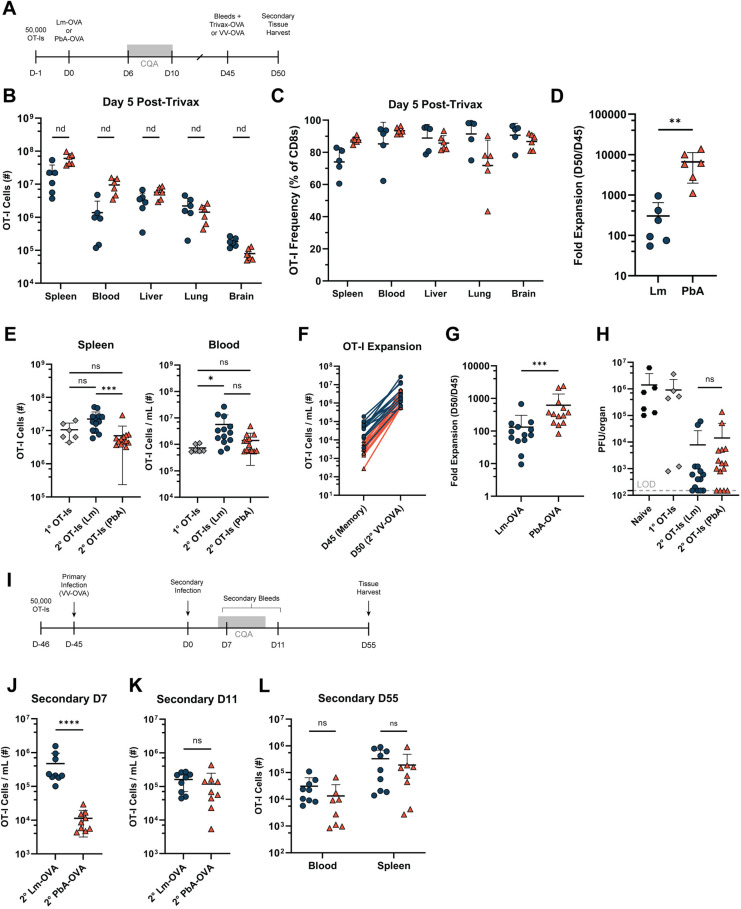
PbA-primed OT-I memory cells expand following secondary stimulus and persist long term following secondary PbA infection. (A) Experimental schematic for B-H: Mice were transferred with OT-I T cells and infected with either Lm-OVA or PbA-OVA. PbA-OVA-infected mice were treated with chloroquine and artesunate on day 6-10 p.i. On day 45 p.i., memory mice were either treated with Trivax-OVA (100 μg agonistic aCD40 mAb, 50 μg OVA peptide, and 50 μg Poly:IC in PBS) or infected with *Vaccinia* virus expressing OVA (VV-OVA). Five days later, the expansion of OT-I T cells was assessed using flow cytometry. (B) Total number of CD45.1^+^ OT-I T cells in the indicated tissues on day 5 post-Trivax-OVA treatment in Lm-OVA (blue) or PbA-OVA (orange) memory mice. Blood represented as # cells/ mL. (C) Frequency of OT-I T cells (of CD8^+^) in the indicated tissues on day 5 post-Trivax-OVA. (D) Fold-expansion of OT-I T cells in the blood on the day of Trivax-OVA treatment and day 5 post-treatment. (E) Number of OT-I T cells in the spleen and blood on day 5 post-secondary infection with VV-OVA in Lm-OVA or PbA-OVA memory mice. A control group receiving naïve OT-I T cells on the day prior to infection was prepared (gray). (F and G) Paired samples (F) and fold-expansion (G) of OT-I T cells following secondary infection with VV-OVA of Lm-OVA and PbA-OVA memory mice. (H) The number of plaque-forming units per mouse ovary are represented. Four groups of mice were tested: naïve control mice (no OT-I T cells), mice transferred with naïve OT-I T cells the day prior to VV-OVA infection, Lm-OVA memory mice, and PbA-OVA memory mice. LOD = limit of detection. (I) Experimental schematic for J-L: Mice were transferred with OT-I T cells and infected with VV-OVA. On day 45 p.i., mice received a secondary infection with either Lm-OVA or PbA-OVA. Secondary bleeds were performed on Day 7 and Day 11 post-secondary infection, and tissues were taken on Day 55. (J and K) Total number of OT-I T cells per mL of blood on Day 7 (J) and Day 11 (K) post-secondary infection with Lm-OVA (blue) or PbA-OVA (orange). (L) Total number of memory OT-I T cells in the blood and spleen on day 55 post-secondary infection. Blood represented as # cells/ mL. Data in A-D is pooled from two independent experiments with **n** = 3 mice per group. Data in B was analyzed with multiple t tests and data in D was analyzed with multiple Mann-Whitney tests. Data in E-H is pooled from three independent experiments with **n** = 2-5 mice per group. Data in E and H were analyzed with a Kruskal-Wallis test. Data in I-L is pooled from two independent experiments with 4-5 mice per group and analyzed with a Mann-Whitney test. *p < 0.05, **p < 0.01, ***p < 0.001, ****p < 0.0001. Error bars are SD.

Next, we tested the ability of the few remaining OT-I memory cells after PbA infection to respond to secondary antigen exposure (Fig 6A). We initially used Trivax [64] immunization (comprised of OVA peptide, Poly(I:C), and agonistic α-CD40 Ab) as a vaccine modality that elicits a strong CD8 T cell response. On day 5 post-immunization, we found a remarkable expansion of OT-I T cells in both groups, with OT-I T cells representing >80% of all CD8 T cells in multiple tissues (Fig 6B & 6C). When comparing the number of OT-I T cells in the blood before and after immunization, OT-I T cells from PbA-OVA-infected mice expanded more than 6,000-fold in just five days, which was significantly more than Lm-OVA infected mice (Fig 6D).

To further test recall potential and functional protection against a viral infection, we challenged Lm-OVA and PbA-OVA memory mice with *Vaccinia* virus expressing OVA (VV-OVA) on day 45 p.i. Five days later, the spleens of mice that were primed with Lm-OVA contained a larger population of OT-I T cells when compared to mice primed with PbA-OVA (Fig 6E). This trend continued in the blood, but was not significant. However, given the diminished size of the OT-I memory pool following PbA infection, these cells again underwent a greater overall expansion (Fig 6F & 6G). We also did not find any differences in the number of VV-OVA plaque-forming units regardless of the primary infection (Fig 6H). Taken together, this data shows that despite the rarity of OT-I T cells after PbA-OVA infection, the remaining memory cells are capable of robust expansion, with comparable viral clearance to OT-I T cells primed with Lm-OVA.

Since the number of memory T cells present can influence the extent of secondary expansion [65,66], we sought to further test expansion on a per-cell basis. We generated memory mice from Lm-OVA and PbA-OVA infection as described above. On day 45 p.i., we sacrificed mice and pooled enriched CD8^+^ CD45.1^+^ cells from each infection cohort. An equal number of memory OT-I T cells (8.5 x 10^3^) were transferred into separate naïve mice (S8A Fig). The following day, these mice were infected with VV-OVA. On day 5 p.i., we found that Lm-primed and PbA-primed OT-I T cells expanded to a similar number in both the blood and spleen (S8B & S8C Fig). These data show that memory OT-I T cells from Lm- or PbA-primed mice are equally capable of expansion following secondary infection.

### Memory OT-I T cells persist long-term following secondary PbA infection

To conclude, we asked whether memory T cells responding to a secondary PbA infection would contract in number similar to naive T cells responding to a primary PbA infection. Mice receiving OT-I T cells were infected with VV-OVA, and on day 45 p.i., mice were given a secondary infection of either Lm-OVA or PbA-OVA (Fig 6I). Consistent with our observations after primary infection, Lm-OVA infection led to a strong increase of OT-I T cells by day 7 post-secondary infection. A secondary stimulus with PbA-OVA peaked on day 11 post-secondary infection (Fig 6J & 6K). Surprisingly, secondary effector OT-I T cells were more resistant to adopting the KLRG1^+^ SLEC phenotype than primary cells (S8D–S8G Fig). Additionally, both groups of mice maintained a large memory population in the blood and spleen up to 50 days post-secondary infection (Fig 6L). These data support the conclusion that memory T cells are more resistant than naïve T cells to PbA-induced modulation of effector differentiation and contraction.

## Discussion

Following an initial burst of proliferation, effector CD8 T cells differentiate into two subsets, influenced by the inflammatory milieu present during priming: KLRG1^+^ IL-7Rα^lo^ SLECs and KLRG1^-^ IL-7Rα^hi^ MPECs. Cytokines such as IL-12 or interferons promote SLEC formation, while limiting these cytokines encourages MPEC formation. Different infections induce a range of SLECs and MPECs among CD8 T cells. LCMV (lymphocytic choriomeningitis virus) and VV (*Vaccinia* virus) induce an intermediate frequency of SLECs (60-70%) during the peak effector phase [4], while vesicular stomatitis virus [7] and influenza [31] induce a lower frequency of SLECs (~30%). During parasitic infection with *Toxoplasma gondii*, KLRG1^+^ cells represent between 40-60% of the responding tetramer-specific CD8 T cells in lymphoid and peripheral tissues [32,33]. Interestingly, blood-stage malaria infection with the less severe *Plasmodium yoelii* strain generates approximately 30% SLECs among the effector CD8 T cell pool [16,67]. In this study, we find that *Plasmodium berghei* ANKA infection remarkably promotes >90% SLEC formation in the blood and peripheral tissues, with only a small quantity of MPECs identified in the spleen.

Following the clearance of PbA parasites using chloroquine and artesunate, memory OT-I T cells are exceedingly rare in the blood and peripheral tissues, especially when compared with mice infected with *Listeria*. Most other infections lose ~90% of their antigen-specific effector T cells during contraction, with 5-10% of cells persisting long term as memory cells [44–48]. In contrast, we found that in mice infected with PbA, less than 1% of effector OT-I T cells persist to memory in the blood. The remaining OT-I T cells are found in lymphoid tissues such as the spleen or lymph node, with very few memory cells found in the blood or peripheral tissues. However, despite limited MPEC formation, most PbA-primed OT-I T cells in the spleen expressed L-selectin, a hallmark of highly proliferative central memory T cells. Thus, robust SLEC formation does not prevent the generation of a diverse memory pool containing central memory T cells. We also found a sizable population of OT-I T cells in the bone marrow following PbA infection. A recent study [68] showed that during dietary restriction, CD8 T cells accumulate in the bone marrow, suggesting that this tissue may be a niche to preserve cells under both metabolic and inflammatory stress.

Based on the striking phenotype of effector OT-I T cells during PbA infection, we suspected that inflammation was a major cause for the diminished memory T cell pool. To this end, we employed several methods to modulate the inflammation present during T cell priming. To initiate malaria-induced inflammation during a known T cell response, OT-I T cells were primed with ΔActA-Lm-OVA, followed by coinfection with wild-type PbA. We found that coinfection with PbA alone was sufficient to reduce memory T cell persistence in both the blood and spleen. These results could have clinical implications for the deployment of vaccines or occurrence of natural infection in environments with regular malaria exposure. To reduce inflammation during PbA infection, we tested whether systemic loss of IFNγ would change the phenotype and persistence of responding OT-I T cells. Loss of IFNγ did significantly improve the formation of MPECs during PbA infection, but this did not affect long-term persistence. Although we found a unique role for PbA-induced inflammation during CD8 T cell differentiation, other factors likely contribute to reduced T cell longevity, and additional research is necessary to fully understand how *Plasmodium* alters effector and memory T cell responses.

We next asked whether PbA-primed memory OT-I T cells could still provide protective immunity during a secondary stimulus. Despite the minimal number of OT-I memory cells in PbA-primed mice, the remaining cells effectively responded to both peptide vaccination and viral infection. The cells were not only highly proliferative, but also functional, clearing VV infection as effectively as Lm-primed OT-I T cells. Given the effector phenotype and subsequent contraction phase measured during primary infection with PbA, we also tested whether memory T cells would be affected in the same manner. OT-I T cells primed by VV-OVA were rested until memory before secondary infection with Lm or PbA. We found that memory OT-I T cells more readily acquire an MPEC phenotype during secondary PbA infection and are much more capable of persistence as compared to naïve T cells. This suggests that naïve CD8 T cells are more susceptible to PbA-induced environmental cues than memory CD8 T cells. Since naïve T cells require both co-stimulation and “signal 3” cytokines for appropriate activation, while memory T cells do not, memory cells may be insulated under some conditions from the negative consequences of a vigorous inflammatory event. Our data also show that despite poor generation of memory cells following PbA, subsequent vaccination or infection is likely to be beneficial and will generate functional effector T cells and enhanced memory cell numbers.

Taken together, we demonstrate here that the quantity and phenotype of circulating CD8 T cells does not necessarily predict the strength of protective immunity provided by a memory T cell population. Furthermore, we find that sampling in the blood did not reflect T cells in the spleen which is a commonly used assumption. These results encourage caution in interpreting human responses to malaria where only blood sampling is routinely evaluated.

Other groups have observed reduced CD8 T cell memory following *Plasmodium berghei* ANKA infection, although this phenomenon has not been investigated mechanistically. Miyakoda et al reported that OT-I T cells were only detectable at memory in 38% of mice infected with PbA-OVA [69]. Ghazanfari et al used a malaria-specific transgenic CD8 T cell rather than OT-I T cells, and found a similar fold reduction (comparing effector and memory T cell populations) to what we report here, although this finding was not discussed [70]. Shaw et al used a different OVA-expressing strain of PbA and infected with 100-fold fewer infected red blood cells, which led to a much more subdued effector OT-I response [35]. However, the number and phenotype of memory OT-I T cells post-PbA infection was consistent with our findings. We add to this research by assessing the phenotype of PbA-primed CD8 T cells during primary infection and the factors contributing to poor memory formation. We report that PbA induces a unique effector differentiation pattern, with a bias toward SLECs at the expense of MPECs. We hypothesize that this phenotype is caused by the inflammatory signature of PbA infection, but further investigations are necessary to identify specific causes beyond IFNγ. We also extended previous findings by showing that PbA-primed OT-I T cells can respond to a secondary challenge, and that these secondary memory cells persist long term.

This data adds to a growing body of evidence demonstrating that malaria infection induces a unique and sometimes dysfunctional immune response. Malaria has been shown to disrupt the architecture of the spleen [71–73], including a study reporting that CD8 T cells responding to malaria may be stranded in the red pulp of the spleen during the effector phase [74]. Similarly, we find that PbA infection induces a greater proportion of cells localized to the red pulp of the spleen. However, whether this localization pattern is detrimental to CD8 T cell function or persistence, or a phenomenon associated with infection of erythrocytes remains to be tested. The disruption of splenic organization caused by malaria also delays germinal center formation, and recent evidence suggests that high levels of systemic inflammation induced by malaria actually impairs the development of T follicular helper (Tfh) cells, collectively leading to a less effective B cell response [61]. Moreover, modulating inflammation during malaria infection via cytokine therapy improves Tfh differentiation and antibody responses [75]. Additionally, it has been shown that hemozoin protein produced by *Plasmodium* parasites compromises dendritic cell function during malaria infection, leading to a reduced humoral immune response [76]. Our findings build upon these studies, demonstrating that PbA programs an effector CD8 T cell response that is almost entirely comprised of SLECs, which appears to limit their ability to persist through contraction. Despite this, we find that memory CD8 T cells retain a remarkable ability to proliferate and perform effector functions upon secondary antigen exposure.

In summary, we show that PbA infection induces a dramatic shift in the effector phenotype of responding CD8 T cells. Effector T cells are comprised almost entirely of SLECs, resulting in a diminished memory pool that is only measurable in select tissues. This phenotype is at least partly caused by PbA-induced inflammation and is partially abrogated in the absence of IFNγ. Consequently, experiencing repeated malaria infections that induce strong inflammatory responses may alter host immunity to both *Plasmodium* and other antigens, and vaccination programs in malaria-endemic countries should consider avoiding periods of high malaria exposure. However, despite the reduced number of memory CD8 T cells generated following blood-stage PbA infection, secondary stimuli with vaccine or infection induces a vigorous T cell response that includes robust effector functions and long-term persistence. Thus, parasitic infections that induce a seemingly poor T cell memory pool can still be beneficial for future host immunity.

## Materials and methods

### Ethics statement

Mice were housed at the University of Minnesota (Minneapolis, MN) and studies were carried out under the Institutional Animal Care and Use Committee approved protocol 2302-40841A. Animal health was monitored daily.

### Mice

All strains used in this publication are on the C57BL/6 background: C57BL/6 and C57BL/6-Ly5.1 (Charles River), OT-I donor mice (JAX #003831, bred in house on the Charles River background), and interferon gamma knockout mice (JAX #002287). All experimental mice, both male and female, were 6-10 weeks old on the date of infection. All mice were housed in specific pathogen-free conditions and bred following all Institutional Animal Care and Use Committee Procedures at the University of Minnesota.

### Microbe strains

*Plasmodium berghei* ANKA wild type (WT) parasites (Dr. Susan Pierce, NIH) and transgenic *Plasmodium berghei* ANKA parasites expressing mCherry-OVA (Dr. Chris Janse, Leiden University Medical Center, Leiden, the Netherlands) parasite lines were passaged in C57BL/6 mice before cryopreservation. Briefly, donor mice were infected with 10^6^ infected red blood cells (iRBCs) from the relevant parasite strain. On day 7 p.i. (parasitemia ~5%), blood from donor mice was diluted 1:2 in Alsever’s solution containing 10% glycerol, then transferred to cryopreservation tubes on dry ice. Vials containing iRBCs were stored in liquid nitrogen, then thawed and diluted in PBS for infection of experimental mice. Recombinant *Listeria monocytogenes* strains Lm-WT (Dr. Hao Shen, U. of Pennsylvania), Lm-OVA (Dr. John Harty, U. of Iowa), and ΔActA-Lm-OVA (Dr. John Harty, U. of Iowa) have been described elsewhere [1]. Bacteria were grown in tryptic soy broth with 50 µg/ml streptomycin to an OD600 of 0.1 before cryopreservation at -80C. Recombinant *Vaccinia*-OVA (Dr. Jonathan Yewdell, NIAID) was stored at -80C, then thawed and diluted into PBS for infection.

### Adoptive transfer of transgenic OT-I T cells

OT-I donor (CD45.1^+^) spleens were mashed through 70 µm cell strainers. Cells were briefly incubated at RT in RBC lysis solution before washing with RPMI supplemented with 5% FBS. CD8 T cells were enriched with the Mojosort Mouse CD8 T Cell Isolation Kit (Biolegend) and collected from the unbound fraction. Enriched CD8 T cells were counted, and a small aliquot of cells were stained with anti-CD8α (clone 53-6.7) and anti-Vα2 TCR (clone B20.1) antibodies to determine purity. Cell counts were adjusted to 5 ^x^ 10^5^ cells/mL in PBS and mice were injected i.v. with 100 µL of cell suspension.

### CRISPR-Cas9 nucleofection

Single guide RNAs (sgRNAs) were purchased from Synthego. The guide RNA sequences used in this study are as follows: sgCD90-1, CAGUCUUGCAGGUGUCCCGA; sgCD90-2, CCGCCAUGAGAAUAACACCA; sgIFNγ-1, AUUUUCAUGUCACCAUCCUU; sgIFNγ-2, UGAAGUCUUGAAAGACAAUC.

Cas9/RNP nucleofection of primary T cells has been previously described [77]. Briefly, OT-I donor (CD45.1+) cells were isolated and sgRNAs for CD90 (sgCD90) or IFNγ (sgIFNγ) were pre-complexed at room temperature for at least 10 minutes. After isolation, OT-I T cells were washed twice with PBS, resuspended in 20uL primary cell nucleofection solution (Lonza), then mixed with the sgRNA/Cas9 complexes. This cell mixture was immediately loaded into a Nucleofection cuvette strip (4D-Nucleofector X kit S; Lonza) followed by electroporation using a 4D nucleofector (4D-Nucleofector Core Unit; Lonza). After nucleofection, prewarmed RPMI + 10% FBS was added to each well, followed by 10 minutes incubating at 37°C. Following incubation, cells were washed once in PBS, counted, then adjusted to 10^6^ cells/mL before being injected i.v. into mice (100μL/mouse).

### *Plasmodium berghei* ANKA infections and quantification of blood parasitemia by flow cytometry

On the day of infection, a vial of PbA or PbA-OVA was thawed and 1x10^6^ iRBCs were injected (i.v.) into experimental mice. Parasitemia was measured by flow cytometry using a previously described method [38] with slight modifications. 1 uL of blood was collected from each mouse via tail snip, followed by fixation in 0.025% glutaraldehyde solution. Cells were washed with PBS, followed by staining with Hoechst dye (RNA stain), dihydroethidium (DNA stain), CD45 (clone 30-F11), and Ter119 (clone TER-119). Samples were collected on a BD Fortessa cytometer and analysis was performed using FlowJo software. Parasitemia was determined as the percentage of red blood cells (Ter119^+^CD45^-^) that stained positive for DNA and RNA.

### *Listeria monocytogenes* infections and determination of CFU

Recombinant *Listeria monocytogenes* strains were thawed from -80°C, then grown in tryptic soy broth containing 50 µg/ml streptomycin to an OD600 of 0.06-0.1. Bacteria were then diluted in PBS before infecting mice. For primary infections with Lm-WT or Lm-OVA, mice were injected with 1-5 x 10^4^ CFU per mouse. Secondary infections with Lm-WT or Lm-OVA used 1 x 10^5^ CFU per mouse. For infections with ΔLm-ActA-OVA, mice were infected with ~2.5 x 10^5^ CFU. The actual CFU injected per mouse was determined by dilution and growth on TSB agar plates containing 50 µg/ml streptomycin.

### *Vaccinia* virus-OVA infections and determination of PFU

Recombinant *Vaccinia* virus expressing OVA was thawed from -80°C. For primary infections, mice were infected with 5 x 10^6^ plaque-forming units of VV-OVA diluted in PBS. For secondary infections, mice were infected with 1 x 10^7^ PFU. Plaque assays for *Vaccinia* virus titration have been described elsewhere, but we used a modified version of Cotter et al [78]. Briefly, ovaries were mashed through 70 μm cell strainers with sterile PBS then stored at -80C before use. Vero cells were plated overnight, then incubated with serial dilutions of VV-OVA. Crystal violet was used to visualize plaques, and experimental samples were compared to stock vials with a known viral titer.

### Antimalarial treatments

To cure mice of blood-stage PbA infection, mice were treated i.p. with chloroquine (30 mg/kg) and artesunate (30 mg/kg) diluted in PBS for five consecutive days starting on day 6 p.i.[35]. Parasitemia was tracked until ~D30 p.i. to ensure elimination of the parasite.

### Restimulation of OT-I T cells with secondary trivax-OVA injection

Trivax [64] was prepared by combining 100 μg aCD40 mAb (BioXCell) + 50 μg OVA peptide (Vivitide) + 50 μg poly:ic (Invivogen) in PBS before i.v. injection into mice.

### Intravascular staining

Staining of intravascular cells has been described previously [29]. Mice were injected retro-orbitally with 3μg of BV421-conjugated anti-CD45 (clone 30-F11). After 3 minutes, mice were sacrificed, and tissues were harvested as described below.

### Tissue processing and antibody staining for flow cytometry

Single cell suspensions were prepared from various tissues as described below. In general, all tissues and cell suspensions were processed using RPMI 1640 (ThermoFisher) containing 5% FBS and 1x penn/strep unless otherwise noted. In some experiments, 1x Brefeldin A (Tonbo Biosciences) was added to processing media to improve direct ex-vivo staining for intracellular markers.

Blood was collected with heparin and treated twice with red blood cell lysis buffer. Spleens and lymph nodes were mashed through 70μm cell strainers, followed by incubation with red blood cell lysis buffer. Bone marrow was collected by cleaning one mouse femur, cutting one end to expose the marrow, followed by brief centrifugation to flush cells from the bone. Livers, lungs, and salivary glands were mechanically dissociated with GentleMACs M tubes (Miltenyi). Lungs and salivary glands were incubated for 1h with 1x collagenase D (Sigma-Aldrich). Livers, lungs, and salivary glands were purified using a 44/67% Percoll gradient. Brains were chopped into 2mm pieces with a razor blade before incubating in 1x collagenase D (Sigma-Aldrich) for 1h. Brains were then purified using a 40/60/90% Percoll gradient.

After processing, cells were resuspended in FACS buffer (PBS with 2% FBS and 0.4% EDTA) and stained with surface markers for 30-60 min at 4°C. Fluorescent dye-conjugated antibodies for the following cell-surface markers were used for staining: anti-CD4 (clone RM4-5), anti-CD8α (clone 53-6.7), anti-CD44 (clone IM7), anti-CD45.1 (clone A20), anti-CD45.2 (clone 104), anti-CD62L (clone MEL-14), anti-CD69 (clone H1.2F3), anti-CD90.2 (clone 30-H12), anti-CD103 (clone M290), anti-IL-7Rα (clone A7R34), anti-CX3CR1 (clone SA011F11), anti-KLRG1 (clone 2F1), anti-Lag3 (clone C9B7W), anti-Tim3 (clone B8.2C12) and anti-PD-1 (clone 29F.1A12). Monomer to identify GAP50^+^ cells (SQLLNAKYL) and OVA^+^ cells (SIINFEKL) were provided by the NIH tetramer core facility and tetramerized with streptavidin-allophycocyanin (APC, Thermo-Fisher) or streptavidin-phycoerythrin (PE, Thermo-Fisher). Samples were then fixed using Cytofix fixation buffer (BD Biosciences).

For intracellular staining, cells were fixed and permeabilized using Cytofix/Cytoperm buffer (BD Biosciences). Intracellular staining for cytoplasmic proteins was performed for 60 min at 4°C using the following antibodies: anti-Granzyme B (clone GB11), anti-IFN-γ (clone XMG1.2), anti-IL-2 (clone JES6-5H4), and anti-CTLA-4 (clone UC10-4B9). For nuclear staining, cells were fixed and permeabilized with the Foxp3 Fixation/Permeabilization Kit (eBioscience) according to the manufacturers’ instructions and stained for 60 minutes at 4°C with anti-Tox1 (clone TXRX10). All samples were collected using a BD Fortessa flow cytometer.

### Data analysis

All flow cytometry data was analyzed using Flowjo software (v10.10). Graphs were created in GraphPad Prism 10 and figures were assembled in Adobe Illustrator.

### Statistical analysis

All data analysis was performed using GraphPad Prism 10. Data was evaluated for statistical significance using a Mann-Whitney test for comparison of two groups, or Kruskal-Wallis test for three or more groups. Unless otherwise stated, *n* represents the number of animals used in an experiment and is stated in the legend for each figure. Data is represented in figures as mean ± standard deviation. A p-value < 0.05 was considered statistically significant.

## Supporting information

S1 Fig OT-I T cells Responding to Severe Malaria Infection are Phenotypically Similar to Endogenous Cells, related to Fig 1. (A) Bar graph showing the number of transgenic OT-I T cells (red) and endogenous GAP50 tetramer^+^ cells (gray) per mouse in the indicated tissues on day 7 post-infection (p.i.) with PbA-OVA. (B) Frequency of intravascular label-positive OT-I T cells and GAP50^+^ cells in the indicated tissues on day 7 p.i. with PbA-OVA. (C) Ratio of IL-7Rα^hi^ KLRG1^-^ memory precursor effector cell (MPEC) phenotype to IL-7Rα^lo^ KLRG1^+^ short-lived effector cell (SLEC) phenotype after gating on either OT-I T cells (red) or GAP50^+^ cells (gray) in the indicated tissues on day 7 p.i. with PbA-OVA. (D) Direct ex-vivo intracellular staining of OT-I (red) and endogenous GAP50^+^ cells (gray) for interleukin 2 (IL-2), interferon gamma (IFNγ), and granzyme B (GzmB) in the indicated tissues on day 7 p.i. with PbA-OVA. (E) Bar graph showing the total number of endogenous GAP50^+^ cells in the indicated tissues on day 45 p.i. Data in A-E is from one independent experiment with *n =* 5 mice and analyzed with multiple unpaired t tests. Data in F is from one independent experiment with *n =* 5 mice. *p < 0.05, **p < 0.01, ***p < 0.001, ****p < 0.0001. Error bars are SD.
(TIF)

S2 Fig Expansion of OT-I T cells in the liver and lungs following infection with Lm-OVA or PbA-OVA, related to Fig 1. (A) Experimental schematic for figs 1 and 2. (B) Representative flow cytometry plots showing identification of OT-I T cells used in all experiments in this manuscript. OT-I T cells expressing CD45.1 were pre-gated as single, live, CD8α^+^, CD44^+^ cells. (C) Frequency, total number, and MPEC:SLEC ratio of CD45.1^+^ cells in the liver on day 7 p.i. with either Lm-OVA (blue) or PbA-OVA (orange). (D) Frequency, total number, and MPEC:SLEC ratio of CD45.1^+^ cells in the lungs on day 7 p.i. with either Lm-OVA or PbA-OVA. (E and F) Representative KLRG1 x IL-7Rα flow cytometry plots showing the effector phenotype of OT-I T cells in the indicated tissues on day 7 p.i. with Lm-OVA (E) or PbA-OVA (F). (G) Ratio comparing the number of OT-I T cells in the brain (left) or lung (right) with the number of OT-I T cells in the spleen on day 7 post-infection. (H) Frequency of intravascular label-positive cells in the spleen on day 7 post-infection with either Lm-OVA (blue) or PbA-OVA (orange). Data in C-G is pooled from three independent experiments with n = 3-5 mice per group and analyzed with a Mann-Whitney test. Data in E-G is pooled from two independent experiments with n = 3-5 mice per group. *p < 0.05, **p < 0.01, ***p < 0.001, ****p < 0.0001. Error bars are SD.
(TIF)

S3 Fig OT-I T cells form a diminished memory pool following infection with PbA-OVA, related to Fig 2. (A) Relative numbers of OT-I T cells in the indicated tissues on day 45 p.i. Gray lines represent the limit of detection by flow cytometry. (B and C) Representative flow cytometry plots and cellularity of CD45.1^+^ OT-I T cells in the indicated tissues on day 45 p.i. with either Lm-OVA (B) or PbA-OVA (C). (D) To establish a “limit of detection” for use in flow cytometry analyses, naïve CD45.1- mice (n=9) were euthanized and tissues were prepared for flow cytometry as normal. Control samples containing CD45.1+ cells were analyzed at the same time and used to draw gates on flow plots. (E) The total number of CD45.1+ cells in the indicated tissues were plotted after analysis and calculations accounting for counting beads and transformation for samples in which a fraction of the tissue was analyzed (for example, 50 uL of blood was the standard amount per sample, and multiplied by 20 to account for number of cells/ mL). The mean number of CD45.1+ events was included above each column, and was used in plots throughout the manuscript. Data in A-C is pooled from three independent experiments with n = 3-5 mice per group and analyzed with a Mann-Whitney U test. Values below the LOD were set to LOD/2 and excluded from phenotypic analysis in S4 Fig. Data in D-E is pooled from three independent experiments with n = 3 mice per group. *p < 0.05, **p < 0.01, ***p < 0.001, ****p < 0.0001. Error bars are SD.
(TIF)

S4 Fig Memory T cell phenotype of OT-I T cells following Lm-OVA or PbA-OVA infection, related to Fig 2. (A and B) Representative KLRG1 x CD62L flow cytometry plots showing the memory phenotype of OT-I T cells in indicated tissues on day 45 p.i. after infection with Lm-OVA (A) or PbA-OVA (B). (C) Frequency of OT-I T cells bearing a KLRG1- CD62L+ central memory (Tcm), KLRG1- CD62L- effector memory (Tem), or KLRG1+ CD62L- long-lived effector cell (LLEC) phenotype in the inguinal lymph node and bone marrow on day 45 p.i. with either Lm-OVA (blue) or PbA-OVA (orange). Other tissues contained too few samples above the limit of detection, and phenotypes were not plotted. (D) Total number of OT-I T cells with a Tcm, Tem, or LLEC phenotype in the inguinal lymph node and bone marrow on day 45 p.i. with Lm-OVA or PbA-OVA. Data in A-D is pooled from two independent experiments with *n* = 3-5 mice per group and analyzed with multiple Mann-Whitney tests. *p < 0.05, **p < 0.01, ***p < 0.001, ****p < 0.0001. Error bars are SD.
(TIF)

S5 Fig PbA-infected mice do not experience lymphopenia or appear exhausted, related to Fig 3. (A-E) The frequency of PD-1^+^, CTLA-4^+^, Lag3^+^, Tim3^+^, or Tox1^+^ cells on the indicated days after infection with Lm-OVA (blue) or PbA-OVA (orange). (F and G) The total number of either bulk CD8+ (A) or bulk CD4+ (B) T cells in the blood in the indicated experimental groups. Data in A and F-G is pooled from three independent experiments with *n* = 3-5 mice per group. Data in B-E is pooled from one independent experiment with n = 5 mice per group. Data in A-E is analyzed with multiple Mann-Whitney U tests *p < 0.05, **p < 0.01, ***p < 0.001, ****p < 0.0001. Error bars are SD.
(TIF)

S6 Fig Phenotype of memory OT-I T cells in IFNγ^-/-^ mice, related to Fig 5. (A-C) The frequency of OT-I T cells bearing a KLRG1- CD62L- effector memory (Tem), KLRG1- CD62L+ central memory (Tcm), or KLRG1+ CD62L- long-lived effector cell (LLEC) phenotype in the spleen on day 45 p.i. with either ΔActA-Lm-OVA or PbA-OVA in WT (blue and orange) or IFNγ^-/-^ (green and yellow) mice. Data in A-C are pooled from three independent experiments with n = 2-4 mice per group. WT and IFNγ^-/-^ mice were compared using a Mann-Whitney test. *p < 0.05, **p < 0.01, ***p < 0.001, ****p < 0.0001. Error bars are SD.
(TIF)

S7 Fig Phenotype of OT-I effector cells after CRISPR-Cas9 deletion of IFNγ, related to Fig 5. (A) Experimental schematic for S7 Fig. OT-I T cells were subjected to CRISPR-based deletion of either CD90 (cCD90, control group) or IFNγ (cIFNγ, experimental group) and rested overnight in either WT or IFNγ^-/-^ mice. The following day, mice were infected with Lm-OVA or PbA-OVA and bled at the indicated effector time points. To measure the efficiency of IFNγ deletion, cells were stimulated with OVA peptide *in vitro* for 5h. (B) Representative histograms showing CD90 staining on cCD90 or cIFNγ OT-I T cells on day 21 p.i. (C) The frequency of CD90+ OT-I T cells between the indicated groups. (D) Representative histograms showing IFNγ staining on cCD90 or cIFNγ OT-I T cells after 5h of *in vitro* OVA peptide stimulation on day 21 p.i. (E) The frequency of IFNγ+ OT-I T cells between the indicated groups. (F-H) The ratio of IL-7Rα^hi^ KLRG1^-^ MPECs to IL-7Rα^lo^ KLRG1^+^ SLECs after gating on CD45.1^+^ OT-I T cells on days 7, 10, 14 p.i. with either Lm-OVA (blue) or PbA-OVA (orange and yellow). Data in B-H is from one independent experiment with n = 3-4 mice per group and analyzed with multiple unpaired t tests. *p < 0.05, **p < 0.01, ***p < 0.001, ****p < 0.0001. Error bars are SD.
(TIF)

S8 Fig Secondary characteristics of OT-I T cells from Lm-primed and PbA-primed mice, related to Fig 6. (A) Lm-OVA and PbA-OVA memory mice were prepared as previously described. On day 44 p.i., the spleens of multiple mice per infection were pooled, enriched, and an equal number of OT-I T cells (8,500) were transferred into separate naïve mice. The following day, mice were secondary infected with VV-OVA, and 5 days later mice were sacrificed to measure the presence of OT-I T cells. (B and C) The number of OT-I T cells in the spleen (B) and blood (C) on day 5 post-infection were plotted after the transfer of an equal amount of memory OT-I T cells. (D and E) The frequency of OT-I T cells bearing a KLRG1+, KLRG1+ IL-7Rα+ (DP), or IL-7Rα+ phenotype on the indicated days after secondary infection with either Lm-OVA (blue) or PbA-OVA (orange). (F and G) Representative KLRG1 x IL-7Rα flow plots showing the phenotype of secondary effector cells on the indicated days post-infection with Lm-OVA or PbA-OVA. Data in A-C is pooled from two independent experiments with n = 5 mice per group and analyzed with a Mann-Whitney test. Data in D-F is pooled from two independent experiments with n = 3-5 mice per group and analyzed with multiple Mann-Whitney tests. *p < 0.05, **p < 0.01, ***p < 0.001, ****p < 0.0001. Error bars are SD.
(TIF)

S1 Data This Excel file contains the source data of all main and supplementary figures. Each sheet in the file contains the data for one figure and is labelled accordingly.
(XLSX)
