# Supplementary figures and images for: Severe malaria enforces short-lived effector cell differentiation but does not prevent effective secondary responses by memory CD8 T cells
Source: PLoS Pathog. 2025 Mar 31;21(3):e1012993. doi: 10.1371/journal.ppat.1012993 (PMC11957282; doi:10.1371/journal.ppat.1012993)

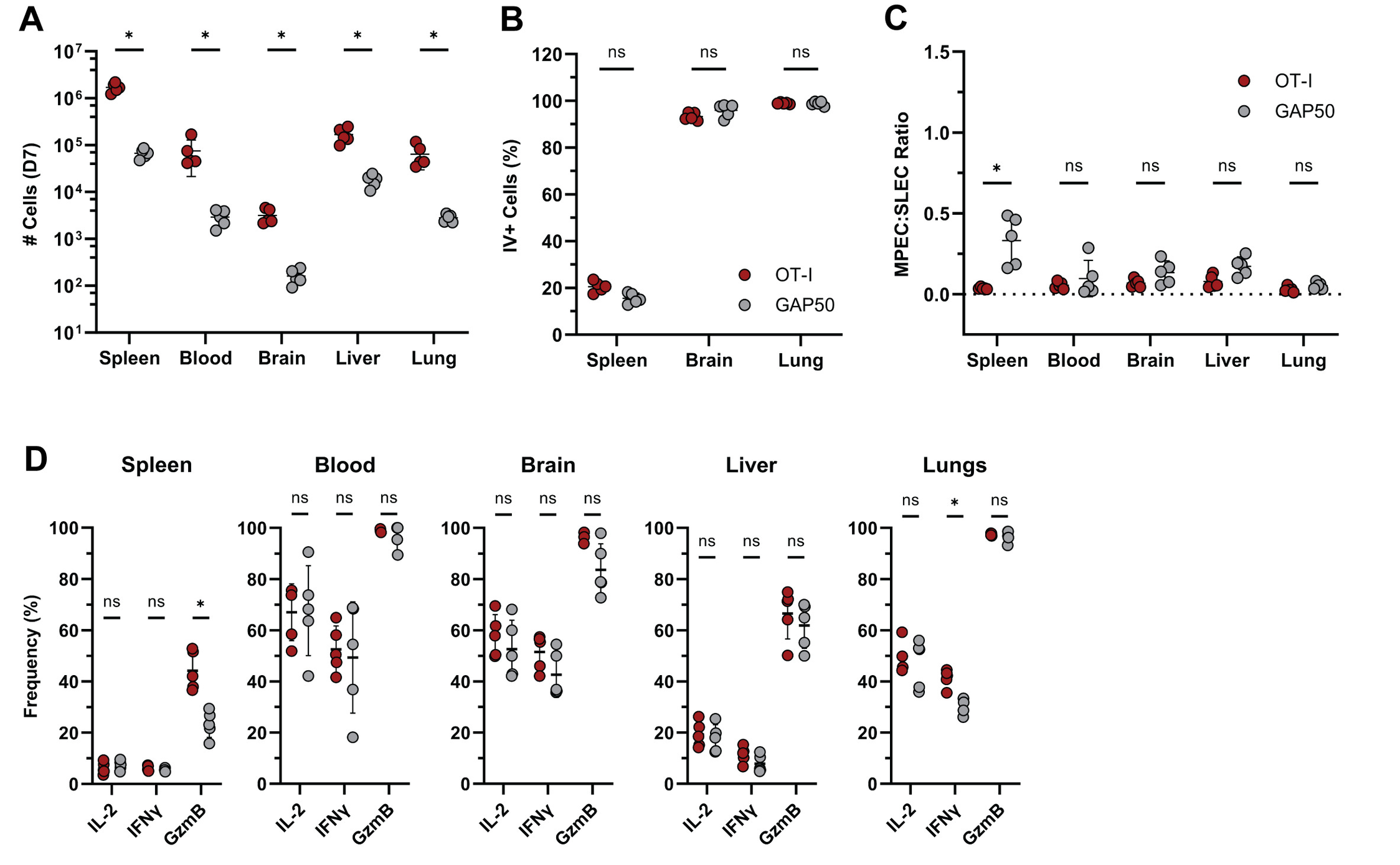

Supplement: S1 Fig — (A) Bar graph showing the number of transgenic OT-I T cells (red) and endogenous GAP50 tetramer+ cells (gray) per mouse in the indicated tissues on day 7 post-infection (p.i.) with PbA-OVA. (B) Frequency of intravascular label-positive OT-I T cells and GAP50+ cells in the indicated tissues on day 7 p.i. with PbA-OVA. (C) Ratio of IL-7Rαhi KLRG1- memory precursor effector cell (MPEC) phenotype to IL-7Rαlo KLRG1+ short-lived effector cell (SLEC) phenotype after gating on either OT-I T cells (red) or GAP50+ cells (gray) in the indicated tissues on day 7 p.i. with PbA-OVA. (D) Direct ex-vivo intracellular staining of OT-I (red) and endogenous GAP50+ cells (gray) for interleukin 2 (IL-2), interferon gamma (IFNγ), and granzyme B (GzmB) in the indicated tissues on day 7 p.i. with PbA-OVA. (E) Bar graph showing the total number of endogenous GAP50+ cells in the indicated tissues on day 45 p.i. Data in A-E is from one independent experiment with n = 5 mice and analyzed with multiple unpaired t tests. Data in F is from one independent experiment with n = 5 mice. *p < 0.05, **p < 0.01, ***p < 0.001, ****p < 0.0001. Error bars are SD. (TIF) [file ppat.1012993.s001.tif]

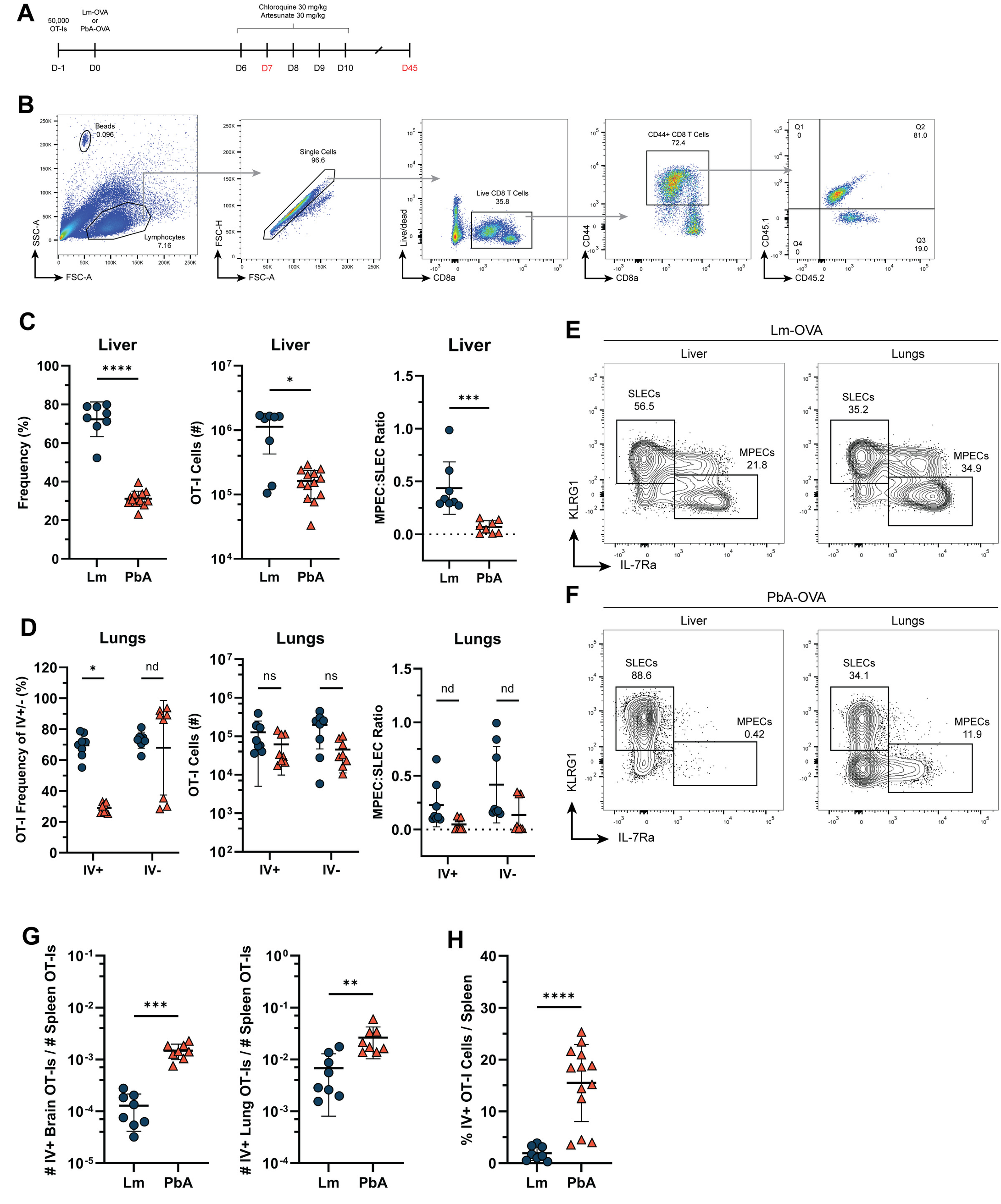

Supplement: S2 Fig — (A) Experimental schematic for figs 1 and 2. (B) Representative flow cytometry plots showing identification of OT-I T cells used in all experiments in this manuscript. OT-I T cells expressing CD45.1 were pre-gated as single, live, CD8α+, CD44+ cells. (C) Frequency, total number, and MPEC:SLEC ratio of CD45.1+ cells in the liver on day 7 p.i. with either Lm-OVA (blue) or PbA-OVA (orange). (D) Frequency, total number, and MPEC:SLEC ratio of CD45.1+ cells in the lungs on day 7 p.i. with either Lm-OVA or PbA-OVA. (E and F) Representative KLRG1 x IL-7Rα flow cytometry plots showing the effector phenotype of OT-I T cells in the indicated tissues on day 7 p.i. with Lm-OVA (E) or PbA-OVA (F). (G) Ratio comparing the number of OT-I T cells in the brain (left) or lung (right) with the number of OT-I T cells in the spleen on day 7 post-infection. (H) Frequency of intravascular label-positive cells in the spleen on day 7 post-infection with either Lm-OVA (blue) or PbA-OVA (orange). Data in C-G is pooled from three independent experiments with n = 3-5 mice per group and analyzed with a Mann-Whitney test. Data in E-G is pooled from two independent experiments with n = 3-5 mice per group. *p < 0.05, **p < 0.01, ***p < 0.001, ****p < 0.0001. Error bars are SD. (TIF) [file ppat.1012993.s002.tif]

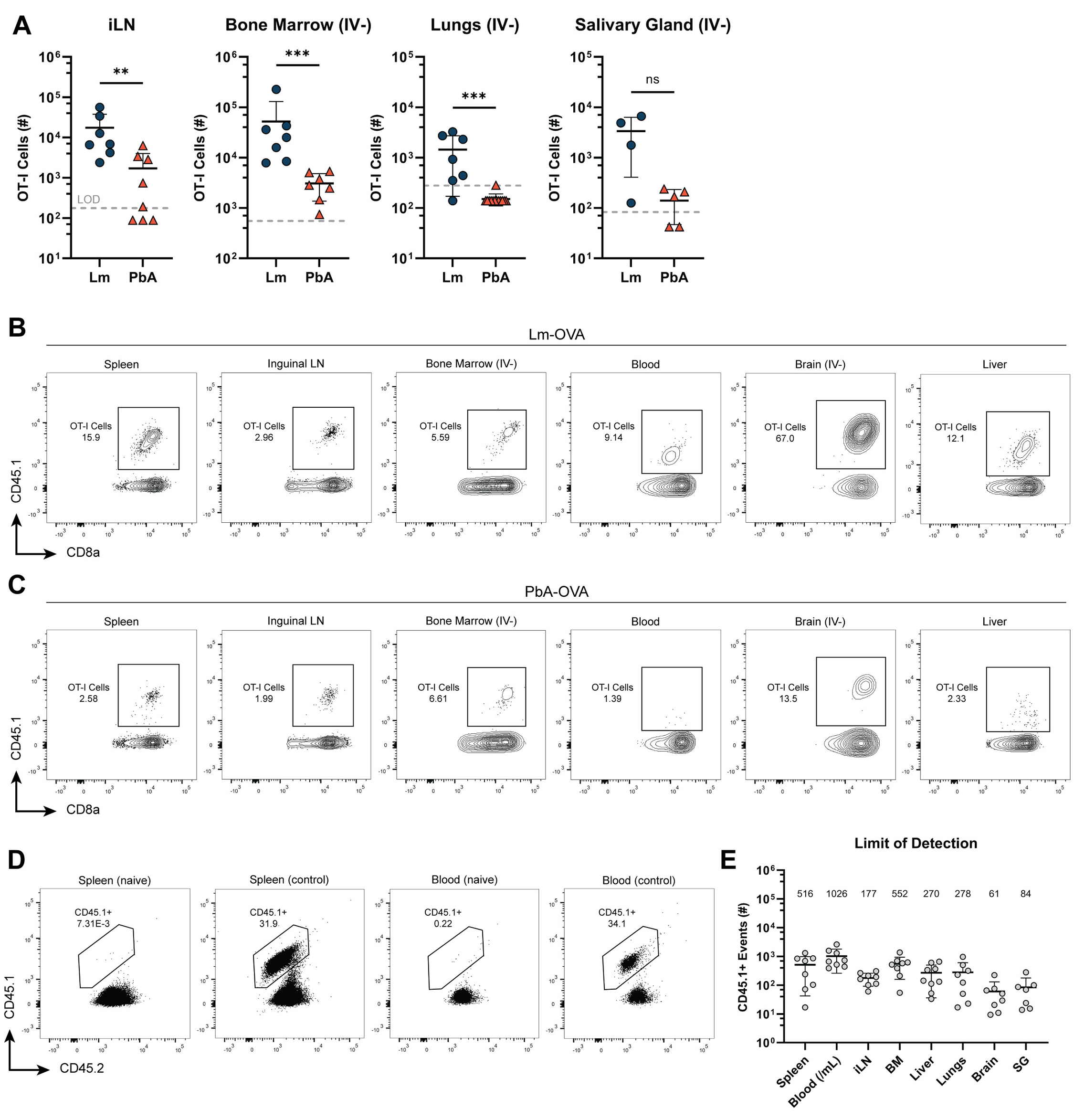

Supplement: S3 Fig — (A) Relative numbers of OT-I T cells in the indicated tissues on day 45 p.i. Gray lines represent the limit of detection by flow cytometry. (B and C) Representative flow cytometry plots and cellularity of CD45.1+ OT-I T cells in the indicated tissues on day 45 p.i. with either Lm-OVA (B) or PbA-OVA (C). (D) To establish a “limit of detection” for use in flow cytometry analyses, naïve CD45.1- mice (n=9) were euthanized and tissues were prepared for flow cytometry as normal. Control samples containing CD45.1+ cells were analyzed at the same time and used to draw gates on flow plots. (E) The total number of CD45.1+ cells in the indicated tissues were plotted after analysis and calculations accounting for counting beads and transformation for samples in which a fraction of the tissue was analyzed (for example, 50 uL of blood was the standard amount per sample, and multiplied by 20 to account for number of cells/ mL). The mean number of CD45.1+ events was included above each column, and was used in plots throughout the manuscript. Data in A-C is pooled from three independent experiments with n = 3-5 mice per group and analyzed with a Mann-Whitney U test. Values below the LOD were set to LOD/2 and excluded from phenotypic analysis in S4 Fig. Data in D-E is pooled from three independent experiments with n = 3 mice per group. *p < 0.05, **p < 0.01, ***p < 0.001, ****p < 0.0001. Error bars are SD. (TIF) [file ppat.1012993.s003.tif]

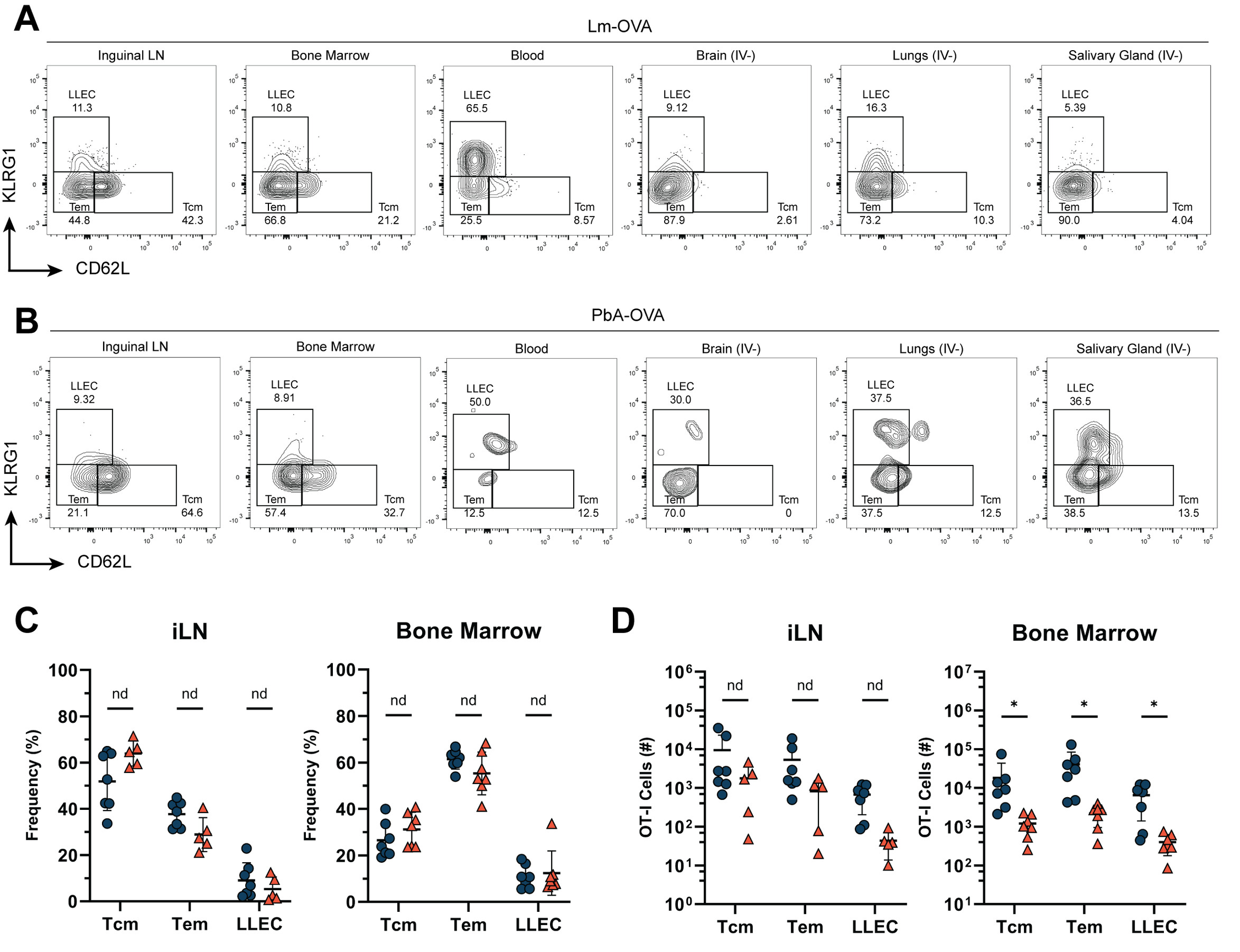

Supplement: S4 Fig — (A and B) Representative KLRG1 x CD62L flow cytometry plots showing the memory phenotype of OT-I T cells in indicated tissues on day 45 p.i. after infection with Lm-OVA (A) or PbA-OVA (B). (C) Frequency of OT-I T cells bearing a KLRG1- CD62L+ central memory (Tcm), KLRG1- CD62L- effector memory (Tem), or KLRG1+ CD62L- long-lived effector cell (LLEC) phenotype in the inguinal lymph node and bone marrow on day 45 p.i. with either Lm-OVA (blue) or PbA-OVA (orange). Other tissues contained too few samples above the limit of detection, and phenotypes were not plotted. (D) Total number of OT-I T cells with a Tcm, Tem, or LLEC phenotype in the inguinal lymph node and bone marrow on day 45 p.i. with Lm-OVA or PbA-OVA. Data in A-D is pooled from two independent experiments with n = 3-5 mice per group and analyzed with multiple Mann-Whitney tests. *p < 0.05, **p < 0.01, ***p < 0.001, ****p < 0.0001. Error bars are SD. (TIF) [file ppat.1012993.s004.tif]

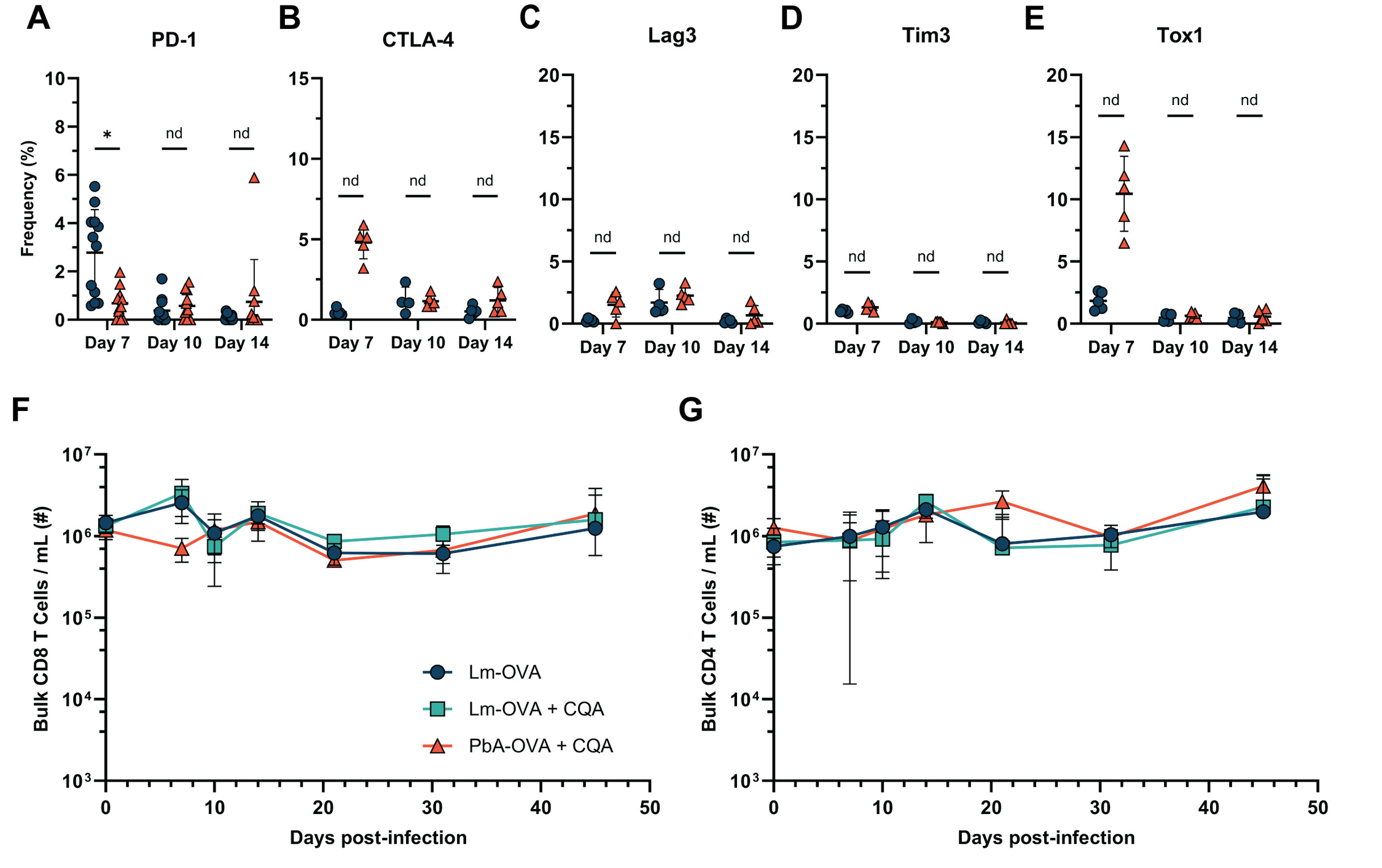

Supplement: S5 Fig — (A-E) The frequency of PD-1+, CTLA-4+, Lag3+, Tim3+, or Tox1+ cells on the indicated days after infection with Lm-OVA (blue) or PbA-OVA (orange). (F and G) The total number of either bulk CD8+ (A) or bulk CD4+ (B) T cells in the blood in the indicated experimental groups. Data in A and F-G is pooled from three independent experiments with n = 3-5 mice per group. Data in B-E is pooled from one independent experiment with n = 5 mice per group. Data in A-E is analyzed with multiple Mann-Whitney U tests *p < 0.05, **p < 0.01, ***p < 0.001, ****p < 0.0001. Error bars are SD. (TIF) [file ppat.1012993.s005.tif]

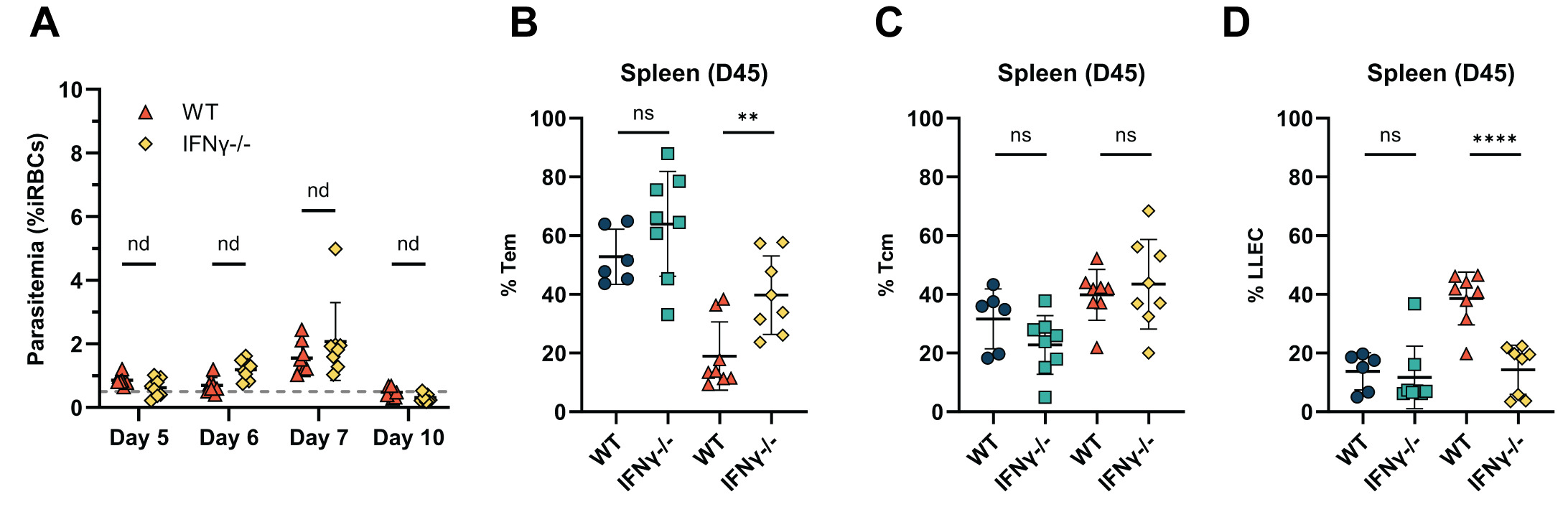

Supplement: S6 Fig — (A-C) The frequency of OT-I T cells bearing a KLRG1- CD62L- effector memory (Tem), KLRG1- CD62L+ central memory (Tcm), or KLRG1+ CD62L- long-lived effector cell (LLEC) phenotype in the spleen on day 45 p.i. with either ΔActA-Lm-OVA or PbA-OVA in WT (blue and orange) or IFNγ-/- (green and yellow) mice. Data in A-C are pooled from three independent experiments with n = 2-4 mice per group. WT and IFNγ-/- mice were compared using a Mann-Whitney test. *p < 0.05, **p < 0.01, ***p < 0.001, ****p < 0.0001. Error bars are SD. (TIF) [file ppat.1012993.s006.tif]

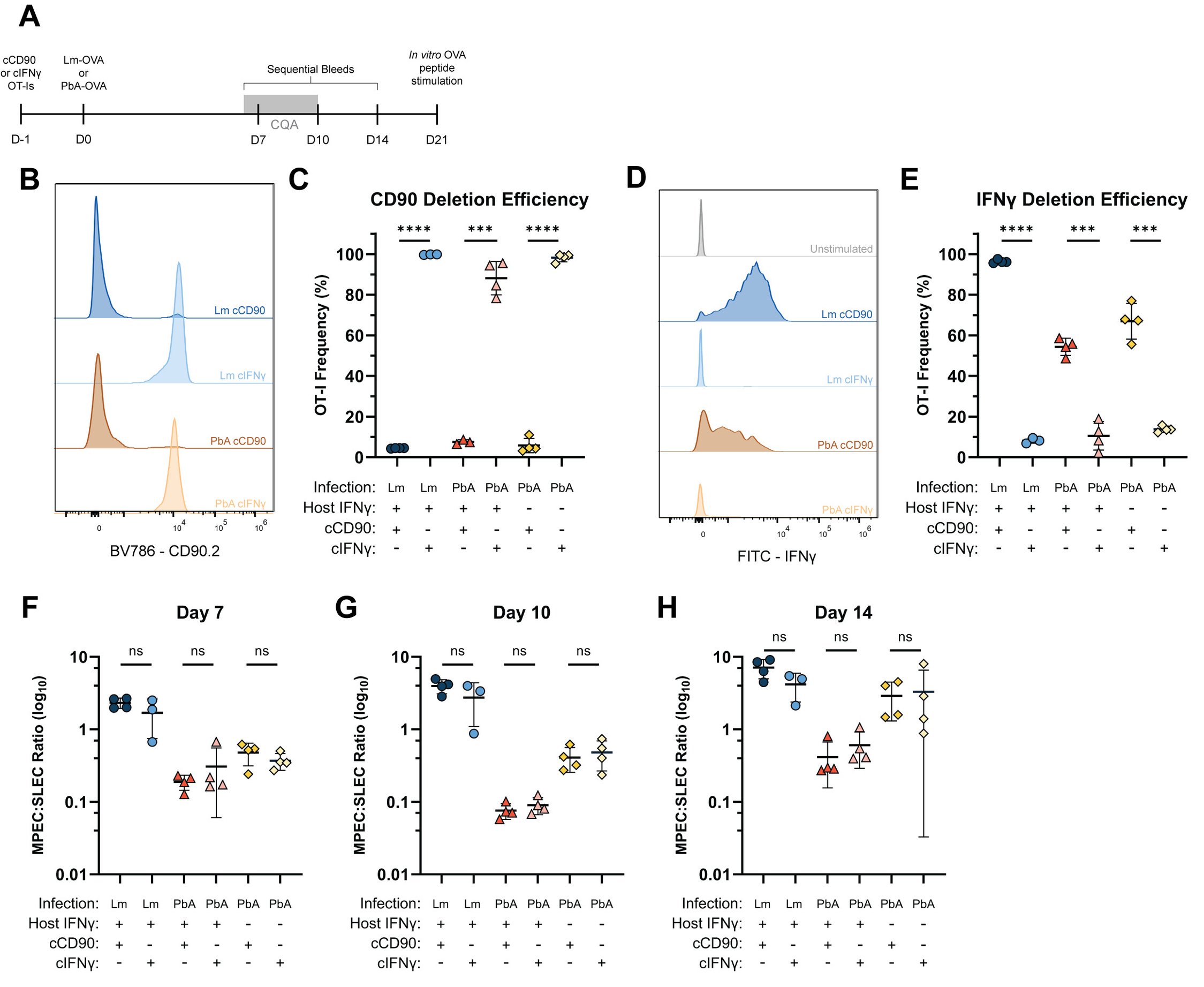

Supplement: S7 Fig — (A) Experimental schematic for S7 Fig. OT-I T cells were subjected to CRISPR-based deletion of either CD90 (cCD90, control group) or IFNγ (cIFNγ, experimental group) and rested overnight in either WT or IFNγ-/- mice. The following day, mice were infected with Lm-OVA or PbA-OVA and bled at the indicated effector time points. To measure the efficiency of IFNγ deletion, cells were stimulated with OVA peptide in vitro for 5h. (B) Representative histograms showing CD90 staining on cCD90 or cIFNγ OT-I T cells on day 21 p.i. (C) The frequency of CD90+ OT-I T cells between the indicated groups. (D) Representative histograms showing IFNγ staining on cCD90 or cIFNγ OT-I T cells after 5h of in vitro OVA peptide stimulation on day 21 p.i. (E) The frequency of IFNγ+ OT-I T cells between the indicated groups. (F-H) The ratio of IL-7Rαhi KLRG1- MPECs to IL-7Rαlo KLRG1+ SLECs after gating on CD45.1+ OT-I T cells on days 7, 10, 14 p.i. with either Lm-OVA (blue) or PbA-OVA (orange and yellow). Data in B-H is from one independent experiment with n = 3-4 mice per group and analyzed with multiple unpaired t tests. *p < 0.05, **p < 0.01, ***p < 0.001, ****p < 0.0001. Error bars are SD. (TIF) [file ppat.1012993.s007.tif]

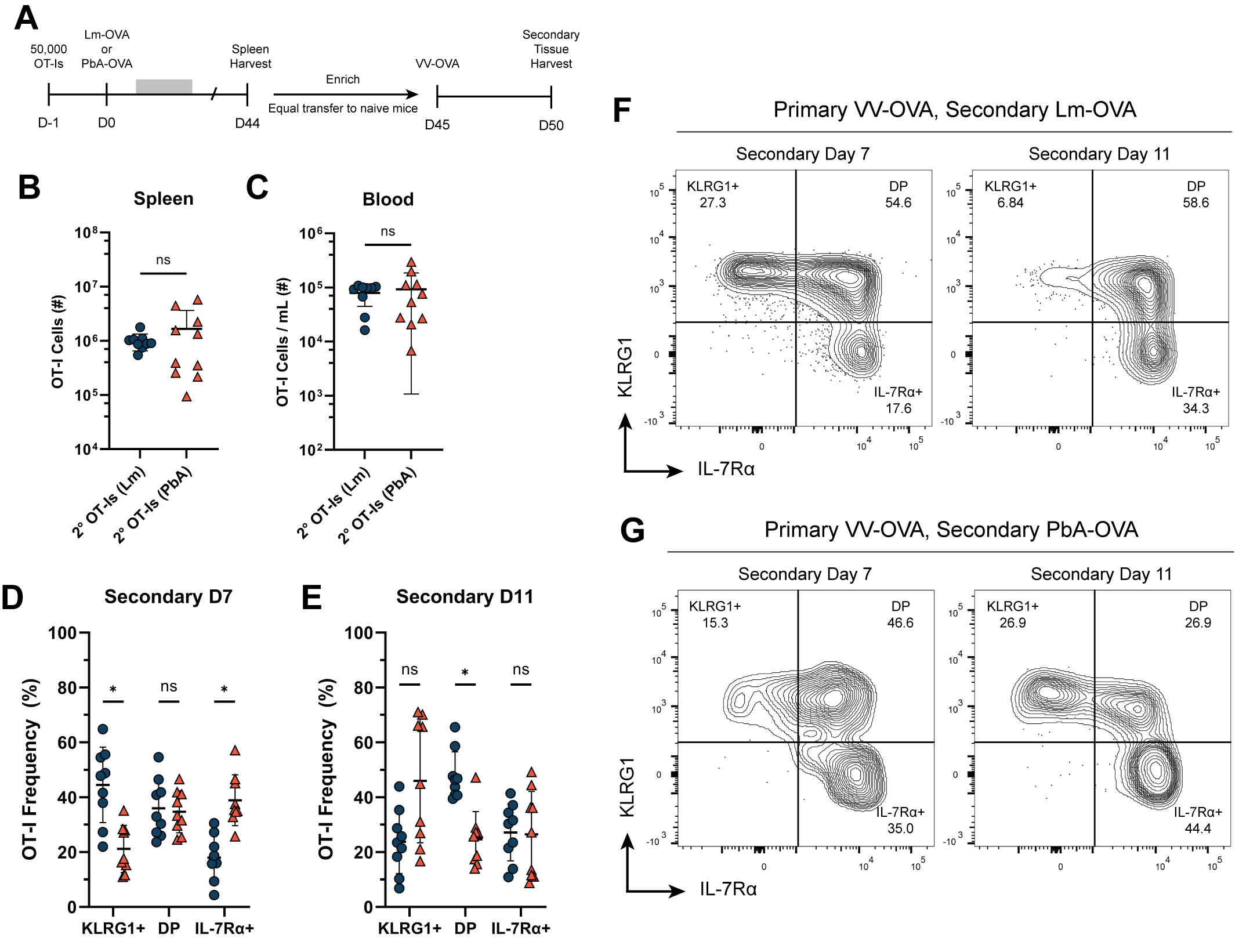

Supplement: S8 Fig — (A) Lm-OVA and PbA-OVA memory mice were prepared as previously described. On day 44 p.i., the spleens of multiple mice per infection were pooled, enriched, and an equal number of OT-I T cells (8,500) were transferred into separate naïve mice. The following day, mice were secondary infected with VV-OVA, and 5 days later mice were sacrificed to measure the presence of OT-I T cells. (B and C) The number of OT-I T cells in the spleen (B) and blood (C) on day 5 post-infection were plotted after the transfer of an equal amount of memory OT-I T cells. (D and E) The frequency of OT-I T cells bearing a KLRG1+, KLRG1+ IL-7Rα+ (DP), or IL-7Rα+ phenotype on the indicated days after secondary infection with either Lm-OVA (blue) or PbA-OVA (orange). (F and G) Representative KLRG1 x IL-7Rα flow plots showing the phenotype of secondary effector cells on the indicated days post-infection with Lm-OVA or PbA-OVA. Data in A-C is pooled from two independent experiments with n = 5 mice per group and analyzed with a Mann-Whitney test. Data in D-F is pooled from two independent experiments with n = 3-5 mice per group and analyzed with multiple Mann-Whitney tests. *p < 0.05, **p < 0.01, ***p < 0.001, ****p < 0.0001. Error bars are SD. (TIF) [file ppat.1012993.s008.tif]
